# Functional Characterization of an *Aspergillus fumigatus* Calcium Transporter (PmcA) that Is Essential for Fungal Infection

**DOI:** 10.1371/journal.pone.0037591

**Published:** 2012-05-23

**Authors:** Taísa Magnani Dinamarco, Fernanda Zanolli Freitas, Ricardo S. Almeida, Neil Andrew Brown, Thaila Fernanda dos Reis, Leandra Naira Zambelli Ramalho, Marcela Savoldi, Maria Helena S. Goldman, Maria Célia Bertolini, Gustavo Henrique Goldman

**Affiliations:** 1 Laboratório Nacional de Ciência e Tecnologia do Bioetanol – CTBE, Campinas, São Paulo, Brazil; 2 Faculdade de Ciências Farmacêuticas de Ribeirão Preto, Universidade de São Paulo, São Paulo, Brazil; 3 Faculdade de Filosofia, Ciências e Letras de Ribeirão Preto, Universidade de São Paulo, São Paulo, Brazil; 4 Faculdade de Medicina de Ribeirão Preto, Universidade de São Paulo, São Paulo, Brazil; 5 Department of Microbiology, University of Londrina, Londrina, Paraná, Brazil; 6 Instituto de Química, UNESP, Araraquara, São Paulo, Brazil; Yonsei University, Republic of Korea

## Abstract

*Aspergillus fumigatus* is a primary and opportunistic pathogen, as well as a major allergen, of mammals. The Ca^+2^-calcineurin pathway affects virulence, morphogenesis and antifungal drug action in *A. fumigatus*. Here, we investigated three components of the *A. fumigatus* Ca^+2^-calcineurin pathway, *pmcA*,*-B*, and *-C*, which encode calcium transporters. We demonstrated that CrzA can directly control the mRNA accumulation of the *pmcA-C* genes by binding to their promoter regions. CrzA-binding experiments suggested that the 5′-CACAGCCAC-3′ and 5′-CCCTGCCCC-3′ sequences upstream of *pmcA* and *pmcC* genes, respectively, are possible calcineurin-dependent response elements (CDREs)-like consensus motifs. Null mutants were constructed for *pmcA* and *-B* and a conditional mutant for *pmcC* demonstrating *pmcC* is an essential gene. The *ΔpmcA* and *ΔpmcB* mutants were more sensitive to calcium and resistant to manganese and cyclosporin was able to modulate the sensitivity or resistance of these mutants to these salts, supporting the interaction between calcineurin and the function of these transporters. The *pmcA-C* genes have decreased mRNA abundance into the alveoli in the *ΔcalA* and *ΔcrzA* mutant strains. However, only the *A. fumigatus ΔpmcA* was avirulent in the murine model of invasive pulmonary aspergillosis.

## Introduction

Calcium ions are extremely important for signal transduction. Two important calcium mediators in the eukaryotic cell are calmodulin and the phosphatase calcineurin [1], [2]. Calcineurin is a heterodimeric protein composed by a catalytic subunit A and a regulatory subunit B [1]. In fungi, calcineurin plays an important role in the control of cell morphology and virulence [1], [2], [3], [4]. The main mode of action of calcineurin is through the dephosphorylation of the transcription factor Crz1p [5]. Calcineurin dephosphorylates Crz1p upon an increase in cytosolic calcium, allowing its nuclear translocation [5], [6]. *CRZ1* deficient mutants display hypersensitivity to chloride and chitosan, a defective transcriptional response to alkaline stress and defects in cellular morphology and mating [5], [7], [8], [9]. Inactivated *Schizosaccharomyces pombe CRZ1* mutants (*Δprz1*) are hypersensitive to calcium and have decreased transcription of the Pmc1 Ca^+2^ pump [10]. *C. albicans* homozygotes *crz1Δ/Δ* display moderately attenuated virulence and sensitive to calcium, lithium, manganese, and sodium dodecyl sulfate [6], [11], [12].

We and others have been characterizing the Ca^+2^-calcineurin pathway in the human pathogenic fungus *A. fumigatus* [3]. In this fungus calcineurin is need for hyphal extension, branching and conidial architecture. Furthermore, the *A. fumigatus ΔcalA* mutant strain has decreased fitness in a low dose murine infection, cannot grow in fetal bovine serum (FBS), and is deficient in inorganic phosphate transport [3], [13]. Three other elements in this pathway were also characterized: (i) the transcription factor CrzA [14], [15], (ii) the RcnA/CbpA, belonging to a class of endogenous calcineurin regulators, calcipressins [16], [17], and (iii) the Golgi apparatus Ca^+2^/Mn^+2^ P-type ATPase PmrA [16]. CrzA mediates cellular tolerance to increased concentrations of calcium and manganese [14], [15]. In addition to acute sensitivity to these ions and decreased conidiation, the *crzA* null mutant suffers from decreased expression of calcium transporters under high calcium concentrations and a loss of virulence. The last identified component of the pathway in *A. fumigatus*, PmrA, has been demonstrated to play a role in cation homeostasis and in the cell wall integrity pathway [16].

Fungal vacuolar Ca^2+^ ATPases are involved in removing Ca^2+^ ions from the cytosol and transporting them to internal stores thus avoiding calcium toxicity [18]). In fungi, the vacuole is a major calcium store and the two main pathways that facilitate the accumulation of Ca^+2^ into vacuoles are the Ca^+2^-ATPases and Ca^+2^/H^+^ exchangers [18]. In *S. cerevisiae*, *PMC1* is responsible for this process preventing growth inhibition by the activation of calcineurin in the presence of elevated calcium concentrations [19]. Here, we report the molecular characterization of three *A. fumigatus PMC1* calcium transporter-encoding genes, *pmcA-C*. We demonstrated that CrzA directly controls the *pmcA-C* mRNA accumulation via binding to their promoter regions. We constructed null mutants for *pmcA-B*, a conditional mutant for *pmcC* and investigated the phenotypes/virulence of these deletions in a murine model of invasive pulmonary aspergillosis. We show that *A. fumigatus pmcC* is an essential gene, while *pmcA* and *pmcB* are both involved in calcium and manganese metabolism. However, only *pmcA* had a dramatic impact on *A. fumigatus* virulence and pathogenicity, since *A. fumigatus ΔpmcA* was avirulent in a murine model of invasive pulmonary aspergillosis.

## Results

### Identification of three *A. fumigatus PMC1* homologues

**Figure 1:**
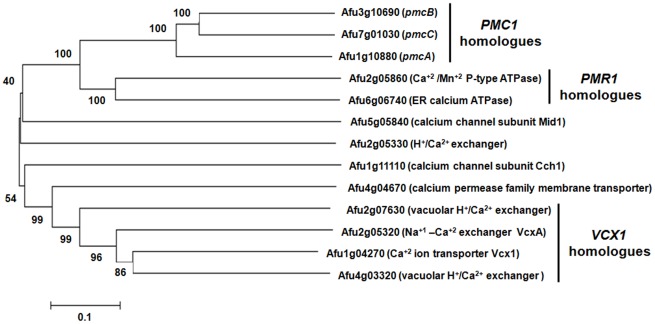
*A. fumigatus* has three *S. cerevisiae PMC1* homologues. Phylogram tree and multiple sequence alignment of calcium transporter orthologues were made in CLUSTAL W2 (http://www.ebi.ac.uk/Tools/clustalw2/index.html) using the default parameters. The followings proteins were used for the analysis: Afu3g10690 (*pmcB*; XP_754550); Afu7g01030 (*pmcC*; XP_746828); Afu1g10880 (*pmcA*; XP_752453); Afu2g05860 (calcium/mangenese P-type ATPase: XP_749715); Afu6g06740 (endoplasmic reticulum calcium ATPase: XP_750567); Afu5g05840 (calcium channel subunit Mid1: XP_754048); Afu2g05330 (vacuolar H+/Ca2+ exchanger: XP_749663); Afu1g11110 (calcium channel subunit Cch1: XP_752476); Afu4g04670 (calcium permease family membrane transporter: XP_746653); Afu2g07630 (vacuolar H+/Ca2+ exchanger: XP_755098); Afu2g05320 (calcium-proton exchanger: XP_749662); Afu1g04270 (calcium ion transporter Vcx1: XP_750174); and Afu4g03320 (similar to vacuolar H^+^/Ca^2+^ exchanger: XP_001481534).

The three main calcium transporters responsible for calcium metabolism in *S. cerevisiae* are *PMC1*, *VCX1*, and *PMR1* [20]. A phylogenetic analysis was performed in order to learn more about homologues of these transporters and other putative *A. fumigatus* calcium transporters (Figure 1). Previously we observed that the mRNA abundance of two *PMC1* orthologous genes, *pmcA* (Afu1g10880) and *pmcB* (Afu3g10690), which encode calcium transporters, was dependent on CalA and CrzA (Soriani *et al.*, 2008). By using this approach, we have identified an additional *PMC1* orthologue, *pmcC* (Afu7g01030). *S. cerevisiae VCX1* encodes a vacuolar antiporter with Ca^+2^/H^+^ and K^+^/H^+^ exchange activity, which is involved in the control of cytosolic Ca^+2^ and K^+^ concentrations [21]. There are four *A. fumigatus* Vcx1p homologues, Afu1g04270 and Afu4g03320 (possibly paralogues), Afu2g07630 and Afu2g05320 (Figure 1). Finally, *S. cerevisiae PMR1* encodes a high affinity Ca*^+^* ^2^/Mn^+2^ P-type ATPase required for Ca^+2^ and Mn^+2^ transport into the Golgi [22]. We have identified two *A. fumigatus PMR1* homologues, Afu2g05860 and Afu6g06740 (Figure 1). Recently, *A. fumigatus pmrA* (Afu2g05860) was characterized [16]. The *ΔpmrA* mutant strain has increased β-glucan and chitin content and it is hypersensitive to cell wall inhibitors, but remains virulent. In addition to these three classes of transporters, we also identified homologues for the calcium channel subunit Mid1 (Afu5g05840), an H^+^/Ca^+2^ exchanger (Afu2g05330), the calcium channel subunit Cch1 (Afu1g11110), and a calcium permease family membrane transporter (Figure 1).

**Figure 2:**
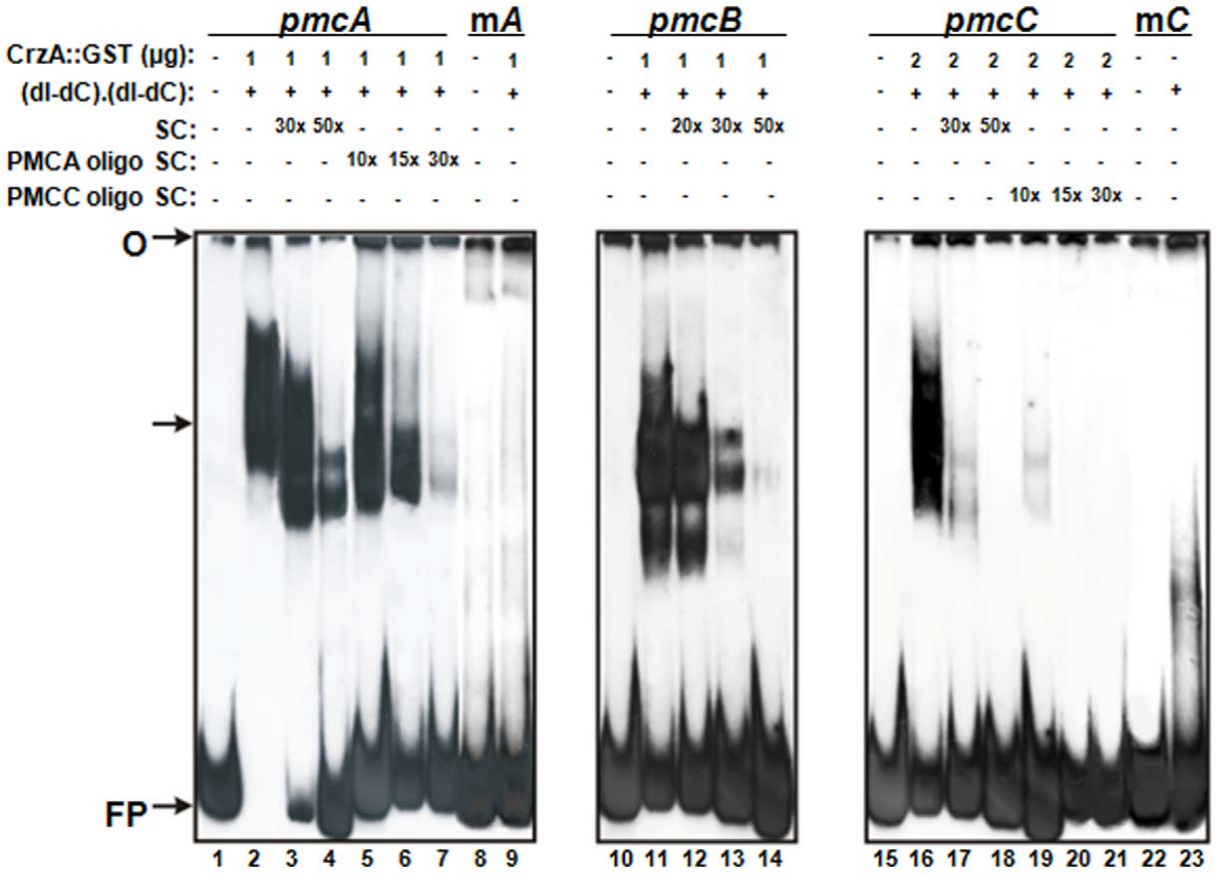
Binding of GST::CrzA recombinant protein to *pmcA-C* promoters. Gel shift analysis was performed using three DNA fragments of *pmcA*, *pmcB* and *pmcC* promoters as probes and 1.0 µg to 2.0 µg of the CrzA*::*GST recombinant protein. Lanes 1 to 7, *pmcA* probe; lane 1, no protein added. Lanes 8 and 9, mutated *pmcA* probe. Lanes 10 to 14, *pmcB* probe; lane 10, no protein added. Lanes 11 to 14, fragments from *pmcB* probe. Lanes 15 to 23, *pmcC* probe; lane 15, no protein added. Lanes 22 and 23, mutated *pmcC* probe. O, gel origin; SC, specific competitor; FP, free probe. The arrow indicates the CrzA*::*GST-DNA complexes.

Here, we concentrate our attention on the molecular characterization of *A. fumigatus PMC1* homologues. These three putative proteins showed approximately 45% identity and 67% similarity (e-value from 7.0e-160 to 1.4e-208) to the *S. cerevisiae PMC1* homologue. PmcA demonstrates 51% identity and 63% similarity with PmcB (e-value 6.9e-271) and 45% identity and 58% similarity with PmcC (e-value 4.e-174) while, PmcB and PmcC showed 53% identity and 66% similarity (e-value 2.7e-276) (for the Clustal aligment of these three proteins, see Supplementary Figure S1). PmcA-C are closely related and probably paralogues (Figure 1). In addition to *pmcA* and *pmcB*, the *pmcC* gene also has decreased mRNA abundance in the *ΔcalA* and *ΔcrzA* mutant strains, respectively, when exposed *in vitro* to CaCl_2_ 200 mM, compared to wild-type *A. fumigatus* [14], [17, data not shown]. To address if CrzA is directly controlling the transcription of *pmcA-C*, we performed Electrophoretic Mobility Shift Assays (EMSA) using purified recombinant GST::CrzA produced in *E. coli*. Previously, we performed an *in silico* analysis using MEME (Motif-based sequence analysis tools; http://meme.sdsc.edu/meme4_1_1/intro.html) to detect the possible presence of a calcineurin-dependent response elements (CDREs)-like consensus motifs in the promoter regions of 28 *A. fumigatus* CrzA-dependent genes [17]. By analyzing their promoter regions, 5′-GT[T/G]G[G/C][T/A]GA[G/T]-3′ was defined as the CDRE-consensus sequence for *A. fumigatus* AfCrzA-dependent genes. When the *pmcA-C* promoter regions (about 500-bp upstream ATG) were scanned for putative CDRE motifs, we were able to identify the 5′-CCCTGCCCC-3′ and 5′-CACAGCCAC-3′ sequences (at −156 and −102 bp from the ATG start codon, respectively) in the *pmcA* and *pmcC* promoter regions. However, we could not identify any conserved CDRE motif in the *pmcB* promoter region (Supplementary Figure S2). Three DNA fragments of about 300-bp located upstream the putative ATG initiation codon of *pmcA-C* genes were used as probes (Supplementary Figure S2). DNA-protein complexes with reduced mobility were observed in the three DNA fragments (Figure 2), however the complexes affinities were different among the three fragments. While 2 µg of GST::CrzA were required for the binding of the *pmcC* probe (Figure 2, lane 16), 1 µg of protein was enough to produce strong DNA-protein complexes for the *pmcA* (Figure 2, lane 2) and *pmcB* (Figure 2, lane 11) probes. This suggests that CrzA has low affinity to the *pmcC* promoter.

The complexes specificities were confirmed by addition of unlabelled probes as specific competitors. Addition of 50-fold molar excess of unlabelled probes completely inhibited the complexes formed with *pmcB* and *pmcC* DNA fragments. The addition of approximately a 15-fold molar excess of the DNA oligonucleotide (5′-CACAGCCAC-3′) inhibited completely the *pmcC* complex specificity (Figure 2, lane 20). However the complex *pmcA-*CrzA was only inhibited in the presence of a 30-fold molar excess of DNA oligonucleotide (5′-CCCTGCCCC-3′) containing the CDRE motif used as specific competitor (Figure 2, lane 7). This result suggests a strong CrzA affinity for this DNA fragment. The specificity of the DNA-protein complex was also confirmed by using mutated *pmcA* and *pmcC* probes, in which the core sequences were changed by site-directed mutagenesis. We have not investigated a mutated *pmcB* DNA fragment because we were not able to identify a conserved CDRE motif in this upstream region. We have not observed the formation of any complex by using both mutated DNA fragments as probes (Figure 2, lane 9 for m*pmcA* probe and lane 23 for m*pmcC* probe). An interesting result was the presence of two complexes exhibiting different molecular masses for *pmcA* and *pmcB* probes. We speculate that they may represent complexes with distinct conformational structures. Additional experiments will be necessary to clarify this. Taken together our results suggest that the mRNA accumulation of *pmcA-C* is directly regulated by CrzA.

### Construction of the *A. fumigatus pmcA-C* mutants

**Figure 3:**
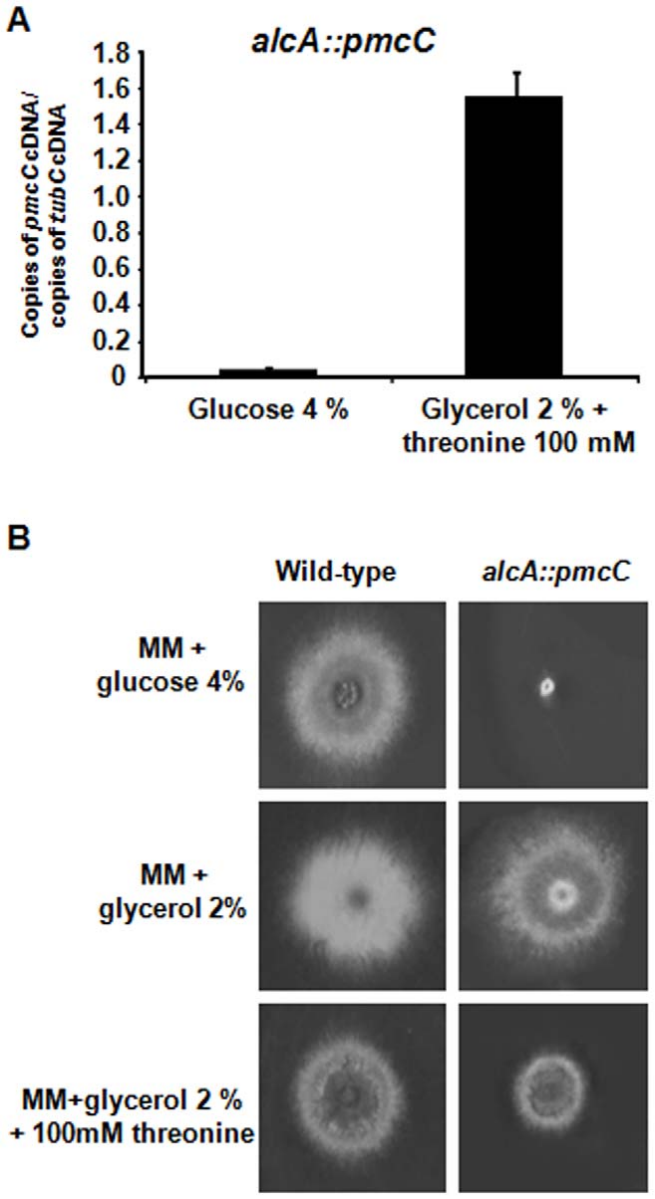
The *pmcC* gene is an essential *A. fumigatus* gene. (A) The *alcA::pmcC* strain was grown for 16 hours in MM+ 2% glycerol at 37°C and transferred into either MM+4% glucose or MM+2% glycerol +threonine 100 mM and grown for further 6 hours. The relative quantitation of *pmcC* and tubulin gene expression was determined by a standard curve (i.e., C_T_ –values plotted against a logarithm of the DNA copy number). The results are the means (± standard deviation) of four biological replicates. (B) Growth phenotypes of the *alcA::pmcC* mutant strain. The *A. fumigatus* wild-type and *alcA::pmcC* mutant strains were grown for 72 hours at 37°C in MM+4% glucose, MM+2% glycerol and MM+2% glycerol +threonine 100 mM.

To get a greater understanding of the role of *pmcA-C*, we tried to inactivate all three genes (Supplementary Figure S3). However, we were unable to inactivate *pmcC*, suggesting that this is an essential *A. fumigatus* gene. Thus, we constructed an *alcA::pmcC* mutant by replacing the endogenous *pmcC* promoter with the *alcA* promoter and verified its growth when the *alcA* promoter was repressed. The *alcA* promoter is repressed by glucose, derepressed by glycerol and induced to high levels by ethanol or L-threonine [23]. We selected a transformant that when transferred from 16 h growth in 2% glycerol, as single carbon source, to 2% glycerol +100 mM threonine for 6 h, the mRNA accumulation of *pmcC* was approximately 15-fold higher than when grown in the presence of 4% glucose (Figure 3A). The repression of *alcA* by growing the *alcA::pmcC* mutant strain in the presence of 4% glucose decreased colony diameter dramatically (Figure 3B). In contrast, both wild-type and *alcA::pmcC* strains demonstrated similar radial diameter when grown in 2% glycerol (Figure 3B). Interestingly, *pmcC* overexpression also decreased the colony diameter size when compared to the wild-type strain, suggesting increased PmcC activity causes some metabolic disturbance that affects growth (Figure 3B). These results strongly indicate *pmcC* is an essential *A. fumigatus* gene.

**Figure 4:**
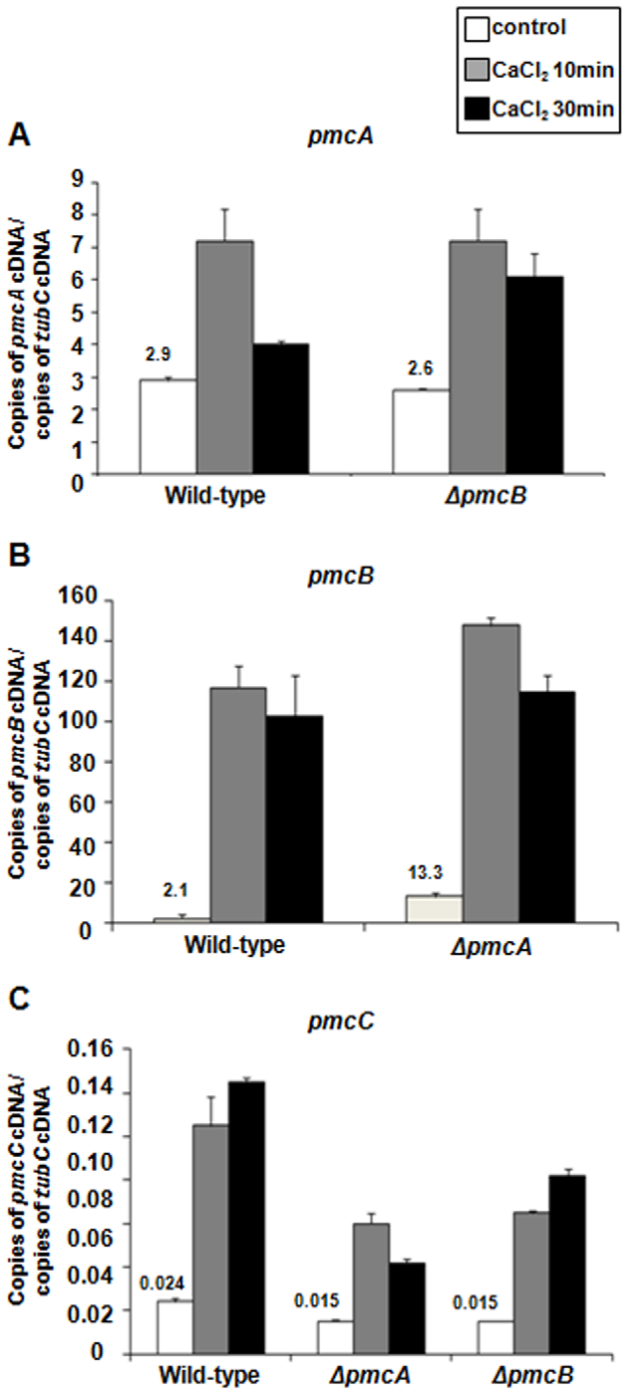
The *pmcA-C* genes have increased mRNA abundance when exposed to calcium. The absolute quantitation of *pmcA*, *pmcB*, and *pmcC* and tubulin gene expression was determined by a standard curve (i.e., C_T_ –values plotted against a logarithm of the DNA copy number). The results are the means (± standard deviation) of four biological replicates. (A–C) The mRNA abundance of *pmcA-C* in the wild-type, *ΔpmcA*, and *ΔpmcB*.

We also compared the absolute levels of mRNA abundance among *pmcA*, *-B*, and *–C* when the *A. fumigatus* wild-type, *ΔpmcA* and *ΔpmcB* mutant strains were exposed to 200 mM CaCl_2_ (Figure 4). Upon exposure of wild-type *A. fumigatus* to calcium, *pmcB* mRNA levels were higher than *pmcA* and *pmcC*, while *pmcA* levels were higher than *pmcC* (Figures 4A–C). The number of normalized *pmcA* and *pmcC* transcripts in the *ΔpmcB* and *ΔpmcA* mutant strains, respectively, were not different from the wild-type strain (Figures 4A and B), suggesting the absence of either *pmcB* or *pmcA* does not considerably affect the mRNA abundance of *pmcA* and *pmcC*. However, before adding 200 mM CaCl_2_ there was approximately six times more *pmcB* transcripts in the *ΔpmcA* than in the wild-type strain (Figure 4B). Interestingly, the *pmcC* mRNA levels are reduced upon CaCl_2_ exposure in both *ΔpmcB* and *ΔpmcA* mutant strains. These results suggest that there is compensation in the mRNA accumulation of *pmcB* in the *ΔpmcA* mutant strain and *pmcC* mRNA accumulation is dependent on *pmcA* and *pmcB*. Upon calcium exposure, down-regulation or overexpression of *pmcC* had no effect on *pmcA* or *pmcB* mRNA accumulation (data nor shown).

**Figure 5:**
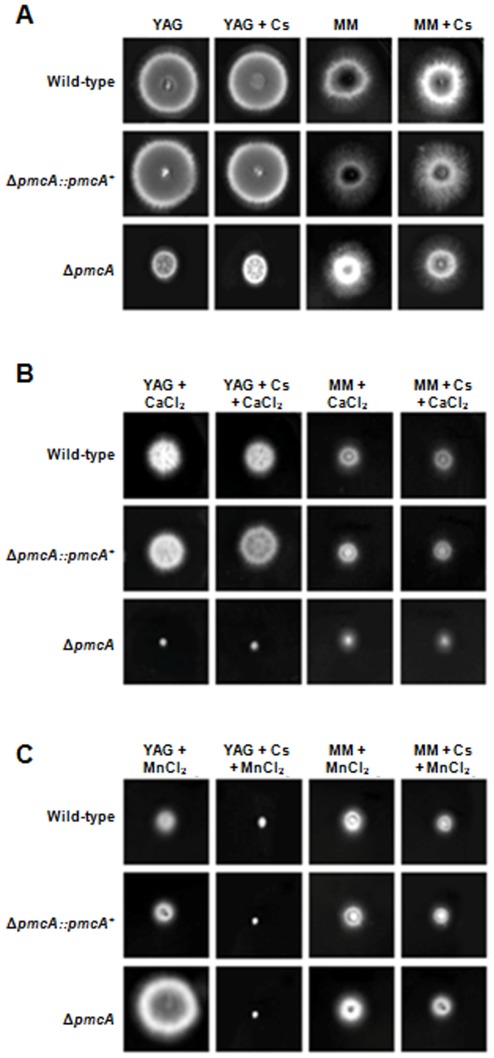
Growth phenotypes of the *ΔpmcA* mutant strain. The *A. fumigatus* wild-type, *ΔpmcA::pmcA* ^+^ and *ΔpmcA* mutant strains were grown for 72 hours at 37°C in (A)YAG, YAG+25 ng/ml cyclosporin (Cs), MM, or MM+25 ng/ml Cs; (B) YAG+500 mM CaCl_2_, YAG+25 ng/ml Cs+500 mM CaCl_2_, MM+500 mM CaCl_2_, or MM+25 ng/ml Cs+500 mM CaCl_2_; (C) YAG+25 mM MnCl_2_, YAG+25 ng/ml Cs+25 mM MnCl_2_, MM+25 mM MnCl_2_, or MM+25 ng/ml Cs+25 mM MnCl_2_.

**Figure 6:**
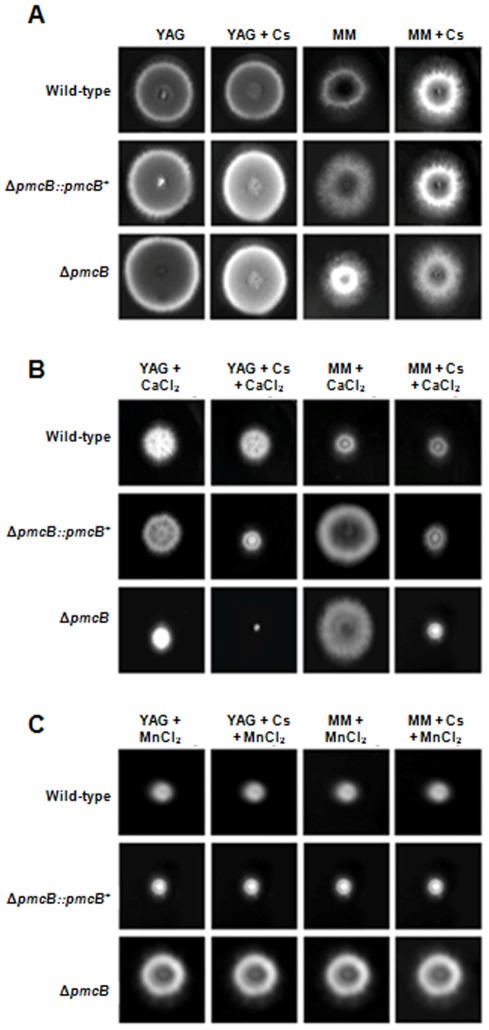
Growth phenotypes of the *ΔpmcB* mutant strain. The *A. fumigatus* wild-type, *ΔpmcB::pmcB* and *ΔpmcB* mutant strains were grown for 72 hours at 37°C in (A)YAG, YAG+25 ng/ml cyclosporin (Cs), MM, or MM+25 ng/ml Cs; (B) YAG+500 mM CaCl_2_, YAG+25 ng/ml Cs+500 mM CaCl_2_, MM+500 mM CaCl_2_, or MM+25 ng/ml Cs+500 mM CaCl_2_; (C) YAG+25 mM MnCl_2_, YAG+25 ng/ml Cs+25 mM MnCl_2_, MM+25 mM MnCl_2_, or MM+25 ng/ml Cs+25 mM MnCl_2_.

Next, we characterized the phenotype of *ΔpmcA* and *ΔpmcB* by growing these strains in different culture media in the presence and absence of cyclosporin A (CsA). This immunosuppressive drug inhibits calcineurin signaling by forming a complex with the immunophilin cyclophilin which then inhibits calcineurin [24]. In addition, since the *ΔcrzA* mutant is also sensitive to MnCl_2_ (Soriani *et al.*, 2008), we decided to investigate a possible influence of *pmcA-B* on this phenotype. Curiously, the *ΔpmcA* mutant strain demonstrated different behavior in complete (YAG) and minimal media (MM) (Figure 5A). It showed reduced radial growth rate in complete medium when compared to the wild-type strain, but this reduction in growth was not suppressed by cyclosporin 25 ng/ml (Figures 5A). The *ΔpmcA* mutant strain was sensitive to CaCl_2_ 500 mM and showed increased sensitivity in YAG and MM, compared to both the wild-type and other mutant strains, when cyclosporin 25 ng/ml was added (Figure 5B). The *ΔpmcA* mutant strain was resistant to MnCl_2_ 25 mM in both YAG and MM media, however cyclosporin suppressed *ΔpmcA* resistance in YAG and wild-type sensitivity in MM (Figure 5C). The *ΔpmcB* mutant strain had about the same radial diameter than the wild-type strain in both MM and YAG media (Figure 6A), but it was much more sensitive to CaCl_2_ in YAG and showed increased sensitivity when grown in the presence of cyclosporin (Figure 6B). However, in MM+500 mM CaCl_2_ the *ΔpmcB* mutant strain has the same radial diameter as the wild-type strain (Figure 6B). In addition, the *ΔpmcB* mutant strain was more resistant to YAG+25 mM MnCl_2_ than the wild-type strain (Figure 6C), but this resistance was suppressed in the presence of cyclosporin (Figure 6C). The same growth was observed for both wild-type and *ΔpmcB* when grown in MM+25 mM MnCl_2_ (Figure 6C). Both the *ΔpmcA::pmcA* ^+^ and *ΔpmcB::pmcB* ^+^ showed the same phenotype as the wild-type strain, strongly indicating that the null phenotypes observed for both genes were only due to the introduction of these mutations in the corresponding strains (Figures 5 and 6).

**Figure 7:**
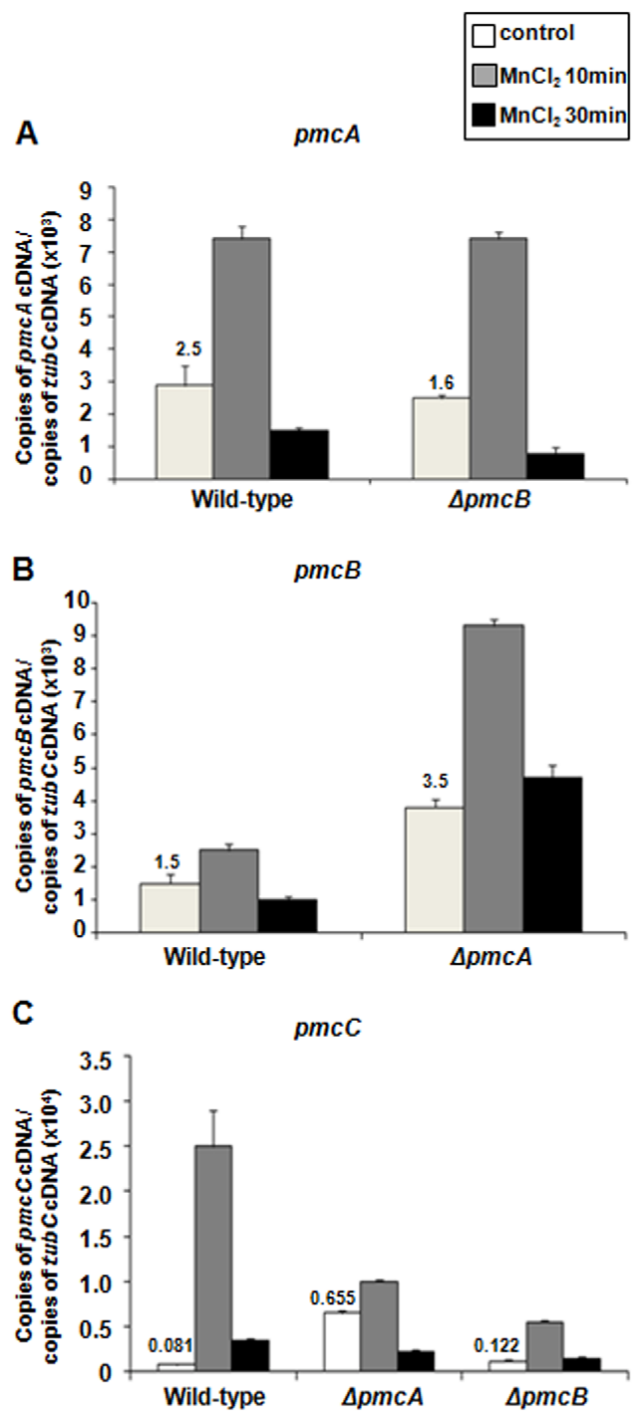
The *pmcA-C* genes have increased mRNA abundance when exposed to manganese. The absolute quantitation of *pmcA*, *pmcB*, and *pmcC* and tubulin gene expression was determined by a standard curve (i.e., C_T_ –values plotted against a logarithm of the DNA copy number). The results are the means (± standard deviation) of four biological replicates. (A–C) The mRNA abundance of *pmcA-C* in the wild-type, *ΔpmcA*, and *ΔpmcB*.

Since the *ΔpmcA* and *ΔpmcB* strains were calcium-sensitive but manganese-resistant, we decide to investigate the mRNA abundance of each *pmc* gene when the wild-type, *ΔpmcA* and *ΔpmcB* mutant strains were exposed to a short pulse of MnCl_2_ (Figure 7). All three genes showed increased mRNA abundance in the presence of MnCl_2_ (*pmcA* and *pmcB* have 2.5- and 2.0-fold more transcripts after 10 minutes; Figures 7A and B), however the highest induction was observed for *pmcC* that showed a 30- and 3.7-fold increase in transcripts after 10 and 30 minutes, respectively (Figure 7C). Nevertheless, like observed for calcium induction, the absolute mRNA levels of *pmcC* are the lowest among all the three genes (Figure 7). The *pmcA* mRNA levels in the *ΔpmcB* mutant strain exposed to MnCl_2_ were about the same as the wild-type strain (Figure 7A). When the *ΔpmcA* mutant strain was exposed to MnCl_2_, the mRNA levels of *pmcB* were 2.5-fold higher than the wild-type strain after 10 minutes exposure. Interestingly, the *pmcB* mRNA levels in this mutant without any MnCl_2_ exposure (*i.e.*, the control before exposure) were 2.3-fold higher than the wild-type strain (Figure 7B). Finally, there was a decrease in the *pmcC* mRNA levels after *ΔpmcA* and *ΔpmcB* mutant strains were exposed to MnCl_2_ (Figure 7C). Down-regulation or overexpression of *pmcC* had no effect on *pmcA* or *pmcB* mRNA accumulation (data nor shown).

**Figure 8:**
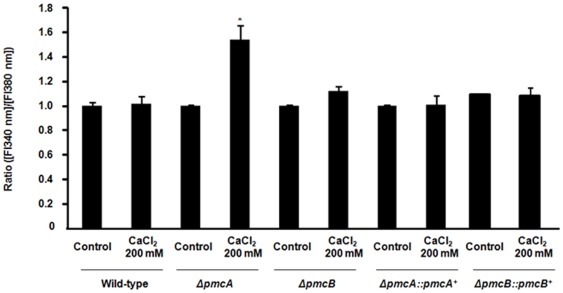
The *ΔpmcA* mutant strain has increased accumulation of calcium in the cytoplasm. The relative levels of intracellular calcium in the wild-type, *ΔpmcA* and *ΔpmcB* mutant strains were determined using the calcium-sensitive dye Fura-2-AM. The relative Ca^+2^ concentration was determined based on the fluorescence ratio after dual-wavelength excitation (fluorescent intensity at 340 nm [FI340 nm]/[FI380 nm]. Data shown are means of three repetitions ± standard deviations. Statistical analysis was performed by using either One-Way Anova with Newman-Keuls post-tests or Tukey's multiple comparison tests. *p<0.005.

Finally, we evaluated the relative concentration of free calcium in the *A. fumigatus* wild-type, *ΔpmcA*, *ΔpmcB*, *ΔpmcA::pmcA* ^+^, and *ΔpmcB::pmcB* ^+^ strains by using Fura-2-AM, a highly sensitive dye for rapid measurement of calcium flux in cells (www.invitrogen.com). Fura-2-Am is a fluorescent calcium indicator that can passively diffuse across cell membranes and when inside the cell, the esters are cleaved by intracellular esterases to yield cell-impermeant fluorescent indicator. Upon binding Ca^+2^, Fura-2 exhibits an absorption shift from 380 to 340 nm of excitation. Thus, the relative Ca^+2^ concentration was evaluated based on the fluorescence ratio after dual-wavelength excitation. Upon calcium exposure, the *ΔpmcA* mutant strain had an increased relative level of intracellular calcium concentration compared to the same strain in the absence of calcium (Figure 8). This difference is not observed for the wild-type, *ΔpmcB* and complemented strains, and *alcA::pmcC* strain (data not shown).

We have not observed any differential susceptibility of these mutants to antifungal agents, such as amphotericin, azoles, and caspofungin, in E-tests (data not shown). These results indicate *pmcA* and *pmcB* are involved in calcium and manganese metabolism in *A. fumigatus*, and also suggest *pmcA* is the major transporter responsible for removing calcium from the cytoplasm.

### Expression of the *pmcA-C* genes in murine-infecting *A. fumigatus* wild-type, *ΔcalA* and *ΔcrzA* mutant strains

Previously, we generated by RNA amplification multiple gene expression profiles via minute samplings of *A. fumigatus* germlings during the initiation of murine infection [25], [26]. This enabled us to identify genes preferentially expressed during adaptation to the mammalian host niche. Here, we took advantage of the establishment of this technical platform to characterize genes that have *in vivo* decreased or increased mRNA abundance in the *ΔcalA* and *ΔczA* mutant strains when compared to the wild-type strain. We firstly characterized the time course of hyphal development in the sequenced clinical isolate Af293, *ΔcalA* and *ΔcrzA* mutant strains by histopathological examination of infected neutropenic murine lung tissues (Supplementary Figure S4). Lung sections collected and formalin-fixed at 4, 10 and 14 hours post-infection contained numerous *A. fumigatus* wild-type, *ΔcalA* and *ΔcrzA* spores in close association with murine epithelium in the bronchioles and alveoli (Supplementary Figure S4, upper panels). At 12–14 hours post-infection, 80% of *A. fumigatus* conidia from the three strains had undergone comparable germination and primary hyphal production. Bronchoalveolar lavage was performed immediately using pre-warmed sterile saline and samples (BALFs) were snap frozen prior to RNA extraction and amplification. Within infection groups BALFs were pooled prior to RNA extraction and mRNA amplification. Total RNA extracted from these cultures was used to amplify fluorescent-labeled cDNAs for real-time PCR experiments. We designed Lux fluorescent probes and used real-time RT-PCR analysis to quantify the *pmcA*, *pmcB*, and *pmcC* mRNA abundance in the *ΔcalA* and *ΔcrzA* germlings after bronchoalveolar lavage at 4 and 14 hours growth and compared this with their expression in the wild-type strain grown during the same time points (reference treatment) (Table 1). All these three genes showed, to different extents, decreased mRNA abundance during the initiation of murine infection by the *A. fumigatus ΔcalA* and *ΔcrzA* mutant strains relative to the wild-type strain. Thus, it seems that *in vivo pmcA-C* mRNA accumulation is dependent on *CalA* and *crzA*.

**Table 1:** Real-time RT-PCR for *pmcA-C* genes from the *in vivo* microarray.

Gene*	Wild-type4 hs	Wild-type14 hs	*ΔCalA*4 hs	*ΔCalA*14 hs	*ΔcrzA*4 hs	*ΔcrzA*14 hs
***pmcA*** **(Afu1g10880)**	0.38±0.01	0.22±0.00	0.20±0.00	0.09±0.00	0.17±0.01	0.11±0.00
***pmcB*** **(Afu3g10690)**	1.52±0.02	3.46±0.23	0.23±0.00	0.32±0.06	0.35±0.08	0.56±0.00
***pmcC*** **(Afu7g01030)**	0.01±0.00	0.01±0.00	0.00±0.00	0.00±0.00	0.00±0.00	0.00±0.00

* The mRNA abundance of *A. fumigatus pmcA-C* genes during growth in lung alveoli. Real-time RT-PCR was used to quantify mRNA abundance. The measured quantity of mRNA for a specific gene in each of the treated samples was normalized using the C_T_ values obtained for the β-tubulin mRNA amplifications run on the same plate. The relative quantitation of a specific gene and β-tubulin gene expression was determined by a standard curve (i.e., C_T_ –values plotted against a logarithm of the DNA copy number). The results of four sets of experiments were combined for each determination; means ± standard deviation are shown. The values represent the cDNA concentration of a specific gene divided by the β-tubulin cDNA concentration.

### The *A. fumigatus ΔpmcA* mutant strain is avirulent in low dose murine infection

**Figure 9:**
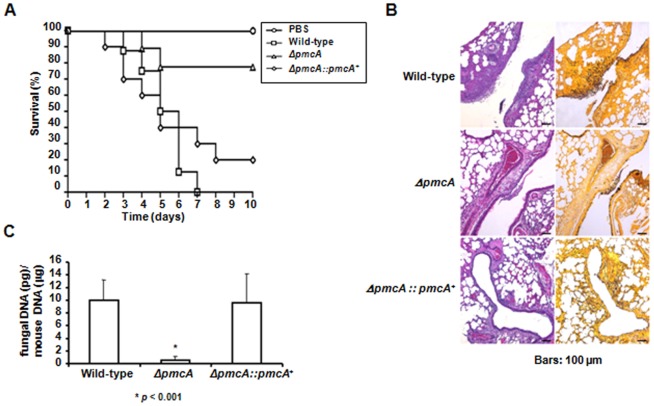
*A. fumigatus pmc*A contributes to virulence in neutropenic mice. (A) Comparative analysis of wild-type, *ΔpmcA* and *ΔpmcA::pmcA* ^+^ strains in a neutropenic murine model of pulmonary aspergillosis. A group of 10 mice per strain was infected intranasally with a 20 µl suspension of conidiospores at a dose of 5.0×10^4^. (B) Histological analysis of infected murine lung were performed 72 hours after infection with the wild-type strain reveals invasion of the murine lung epithelium (C) Fungal burden was determined 48 hours post-infection by real-time RT-PCR based on 18S rRNA gene of *A. fumigatus* and an intronic region of the mouse GAPDH gene. Fungal and mouse DNA quantities were obtained from the Ct values from an appropriate standard curve. Fungal burden was determined through the ratio between ng of fungal DNA and µg of mouse DNA quantities. The results are the means (± standard deviation) of five lungs for each treatment.

To assess the role of PmcA-B in pathogenicity we tested the *A. fumigatus ΔpmcA-B* mutant strains in a neutropenic murine model of invasive pulmonary aspergillosis, comparing virulence of the *A. fumigatus ΔpmcA-B* mutant strains (n = 10 for each mutant) to that of the wild-type (n = 10) (Figure 9A and Supplementary Figure S5). While infection with the wild-type strain resulted in a mortality rate of over 100% at 6 days post-infection, infection with the *pmcA* deletion strain resulted in a significantly reduced mortality rate of approximately 20% after 10 days post-infection (p<0.005). The *pmcB* mutant showed virulence comparable to the wild-type strain (Supplementary Figure S5). Since the comparison between *ΔpmcA* infected group and the non-infected group (PBS) showed to be statistically non-significant (*p* = 0.1451), we can consider this strain avirulent. To directly link the observed attenuated virulence of the *ΔpmcA* mutant with the replacement of the *ΔpmcA* locus we tested an independent strain resulting from single ectopic reintegration of the wild-type *ΔpmcA* locus (Supplementary Figure S6) and with the complementation strain full virulence was restored (Figure 9A). To further understand the basis of attenuated virulence in the *ΔpmcA* background we made histopathological examinations of infected tissues at early time points in infection, aiming to identify differences in growth rate, tissue invasion and inflammatory responses between the two strains. At 72 hours post-infection the lungs of mice infected with the wild-type isolate contained multiple foci of invasive hyphal growth, manifesting as both penetration of the pulmonary epithelium in major airways (Figure 9B) and pockets of branched invading mycelia originating from the alveoli (Figure 9B). In contrast, infection resulting from *ΔpmcA* inoculations was typified by contained inflammatory infiltrates in bronchioles (Figure 9B) some of which contained fungal elements in the form of poorly germinated or ungerminated spores. Fungal burden data as measured by real-time PCR showed that the *ΔpmcA* mutant strain did not grow within the lungs as well as the wild-type and the complemented *ΔpmcA* strains (Figure 9C, *p*<0.0001). Taken together, these data strongly indicate that PmcA plays a role in *A. fumigatus* virulence.

## Discussion

We have been actively looking for additional components of the Ca^+2^-calcineurin pathway [14], [17], [27]. One of these components, the transcription factor CrzA induces the expression of various cation transporters that act at the plasma membrane or on other membranous organelles [14], [17]. Very little is known about calcium transport and calcium homeostasis in filamentous fungi. Most of our knowledge about calcium homeostasis in fungi is derived from *S. cerevisiae*, where more than 95% of cellular calcium is sequestered in the vacuole [28], [29], [30]. In *S. cerevisiae PMC1*, *PMR1*, and *VCX1* encode a vacuolar Ca^2+^ ATPase involved in depleting cytosol of Ca^2+^ ions, a high affinity Ca^2+^/Mn^2+^ P-type ATPase required for Ca^2+^ and Mn^2+^ transport into the Golgi, and a vacuolar membrane antiporter with Ca^2+^/H^+^ and K^+^/H^+^ exchange activity, involved in the control of cytosolic Ca^2+^ and K^+^ concentrations, respectively [19], [22], [31]. *S. cerevisiae PMC1* knock-out mutants sequester Ca^+2^ into the vacuole at 20% of the wild-type levels and fail to grow in media containing high levels of Ca^+2^ [19]. Mutations in the calcineurin A or B subunits or the addition of FK506 or cyclosporin A restored growth of *pmc1* mutants in media with high Ca^+2^ concentrations [20], [21]. In *Neurospora crassa* it was reported that active transport across the plasma membrane is important for keeping low levels of cytosolic calcium [32], [33]. In addition, in this species the vacuole is important for regulating the intracellular calcium levels [32], [34], [35]. In *N. crassa*, there are two *PMC1* homologues, *NCA-2* and *NCA-3* [36]. The *NCA-2* fused with GFP is located in the plasma membrane as well as in vacuolar membranes in this organism [37], suggesting *NCA-2* functions to pump calcium out of the cell. The *Δnca-3* strain showed comparable levels of calcium sensitivity to the wild-type strain; in contrast, *Δnca-2* showed significant inhibition of growth at 50 mM CaCl_2_ and accumulates 10-fold more intracellular calcium than the wild-type strain [36]. In *A. nidulans*, two null mutants constructed for the *PMC1* homologues, *pmcA* and *pmcB*, displayed low-sensitivity to 700 mM CaCl_2_ concentrations [38]. However, the double *A nidulans ΔpmcA ΔpmcB* mutant has increased calcium-sensitivity suggesting these two genes are genetically interacting [38].

Rispail *et al.* [39] have proposed that *A. fumigatus* has three *PMC1*, two *PMR1*, and four *VCX1* homologues. Here, we have concentrated our attention on three genes encoding *PMC1* calcium transporter homologues that have their mRNA levels dependent on CalA-CrzA [14], [17], generated mutants of them, and studied their phenotypes and virulence. Although *A. fumigatus pmcA-C* genes are involved in calcium metabolism, this work did not provide a full characterization of their function. We do not know their sub-cellular localization and how they affect sub-cellular calcium abundance. We were not able to knock-out *pmcC* and subsequently demonstrated that *pmcC* is an essential *A. fumigatus* gene by constructing a conditional *pmcC* mutant. The *pmcC* downregulation causes growth inhibition and its overexpression can produce a physiological imbalance, as the mutant strain also has reduced growth. We were able to demonstrate that CrzA can control the *pmcA-C* mRNA expression by binding directly to their promoter regions. Crz1p has a C2H2 zinc finger motif that binds to CDRE in the promoters of genes that are regulated by calcineurin and calcium (Stathopoulos and Cyert, 1997). Yoshimoto *et al.* [40] have identified the *S. cerevisiae* Crz1p-binding site as 5′-GNGGC(G/T)CA-3′ by *in vitro* site selection. Recently, Hagiwara *et al.* [41] identified and characterized the *A. nidulans* An*crzA* gene. They performed an *in silico* analysis by also using MEME of the possible presence of a CDRE-like consensus motif in the promoter regions of 25 AnCrzA-dependent genes. By analyzing their promoter regions, 5′-G[T/G]GGC[T/A]G[T/G]G-3′ was presumed to be the consensus sequence for the *A. nidulans* AnCrzA-dependent genes. By using a combination of MEME analysis and the *A. nidulans* CDRE consensus as a guide, we were able to identify 28 *A. fumigatus* genes that were repressed in *ΔcrzA* mutant strain upon CaCl_2_ exposure (Soriani *et al.*, 2010), with 5′-GT[T/G]G[G/C][T/A]GA[G/T]-3′ as the CDRE-consensus sequence for *A. fumigatus* AfCrzA-dependent genes. Here, we demonstrated that CrzA can bind directly to 300-bp upstream regions from *pmcA-C* genes. In two of these genes, *pmcA* and *pmcC*, we were able to identify putative CDRE motifs and demonstrated that they can completely inhibited the complexes formed with *pmcA* and *pmcC* DNA fragments. These results strongly suggest these CDRE motifs are functional and this is probably the first demonstration of CDRE functionality in a human pathogenic fungus.

Cyclosporin was able to modulate the sensitivity or resistance of these mutants to either calcium or manganese chloride, once more supporting the interaction between calcineurin and the function of these transporters. In addition, we showed wild-type levels of susceptibility to amphotericin B, voriconazole, posoconazole, itraconazole, and caspofungin (E-test assays) and that there were no defects in cell wall integrity (data not shown). We also observed that the complete and minimal culture media affected the susceptibility of the *ΔpmcA* and *ΔpmcB* mutant strains to calcium and manganese chloride. The defined macronutrients composition of MM medium could explain the differences in growth of the *ΔpmcA* and *ΔpmcB* mutant strains in YAG and MM media. The MM is composed of glucose, trace elements, and macronutrients (salt solution). The salt solution is composed of sodium nitrate, potassium chloride, potassium phosphate, and magnesium sulphate. When the wild-type, *ΔpmcA*, and *ΔpmcB* strains are grown in MM supplemented only with a single one of these macronutrients, there is a reduction in radial growth for all strains, except for MM+MgSO_4_ that showed about the same radial growth as in MM (data not shown). The most likely reasons for this outcome are either the mechanism of action of these transporters depends on other cations, such as sodium, or there is some cross-talk with the mechanisms for ion detoxification. Recently, Spielvogel *et al.* [42] have shown that SltA, a transcription factor important for cation adaptation and homeostasis acts positively on the transcription of the Ena1p-like Na^+^ pump gene *enaA* and negatively on the transcription of the putative vacuolar Ca^+2^/H^+^ exchanger gene *vcxA* (*A. fumigatus* homologue is Afu1g04270). Interestingly, the negative regulation of *vcxA* by SltA is opposed by its transcriptional activation by CrzA [42].

*A. fumigatus pmcA-C* genes have decreased mRNA abundance into the alveoli in the *ΔcalA* and *ΔcrzA* mutant strains. Accordingly, when *A. fumigatus* is exposed *in vitro* to calcium chloride, there is a decrease in *pmcA-C* mRNA abundance in both mutants. When we compare the absolute *pmcA-C* mRNA abundance levels in the wild-type strain grown in mouse alveoli, we observed that *pmcB* has about five to ten times higher levels than *pmcA*, while *pmcC* has very low levels of mRNA abundance (1,000 to 3,000 times lower than *pmcB*). Consistently, the same mRNA abundance is observed when *A. fumigatus* is exposed *in vitro* to calcium chloride. Interestingly, there is an increase in the *pmcB* mRNA levels in the *ΔpmcA* mutant strain when this strain is not exposed to CaCl_2_, suggesting a compensation for the *pmcA* absence. An intriguing observation from our work is the fact that *pmcC* has very low absolute levels of mRNA accumulation in all conditions tested in this work, but it is an essential gene. This is confirmed by a weak CrzA binding to *pmcC* promoter. It is possible that PmcC specific activity is very high and this will compensate its low mRNA levels. It is also possible that PmcC has other functions that were not identified in this work and are essential for cell metabolism. Interestingly, both *ΔpmcA* and *ΔpmcB* mutants are more resistant to MnCl_2_ than the wild-type strain and had reduced *pmcC* mRNA accumulation when exposed to either CaCl_2_ or MnCl_2_. These results suggest *pmcC* mRNA levels are dependent on *pmcA* and *pmcB*, when *A. fumigatus* is exposed either to calcium or manganese. However, this effect is more notable in the presence of manganese.

The *ΔpmcA* mutant is avirulent in a neutropenic murine model of invasive pulmonary aspergillosis. The reduced virulence of the *ΔpmcA* could be due to an excess of calcium in the cytoplasm that could not be removed due to the lack of *pmcA*, thus potentially affecting several functions such as secretion, cell wall composition and the activation of pathways necessary for infection. Interestingly, we did not observe attenuated virulence for *ΔpmcB*, suggesting that the different *PMC1* paralogues have different functions during pathogenicity. This is the first demonstration of the involvement of a calcium transporter in *A. fumigatus* virulence. Previously, Pinchai *et al*. [16] have shown that *A.fumigatus ΔpmrA* has several defects related to growth, cationic tolerance, and increased beta-glucan and chitin content, but in spite of all these abnormal phenotypes the mutant strain remained virulent.

In conclusion, we have shown that PmcA is required for full virulence in animal infection. In addition, that PmcA acts in the *A. fumigatus* Ca^+2^-calcineurin signaling pathway and influences the relative intracellular calcium concentration. Further studies are necessary to address the sub-cellular location of PmcA, -B, and –C, and how PmcA contributes to the pathogenesis of aspergillosis.

## Materials and Methods

### Ethics statement

The principles that guide our studies are based on the Declaration of Animal Rights ratified by the UNESCO in January 27, 1978 in its articles 8^th^ and 14^th^. All protocols used in this study were approved by the local ethics committee for animal experiments from the Campus of Ribeirão Preto from Universidade de Sao Paulo (Permit Number: 08.1.1277.53.6; studies on the interaction of *Aspergillus fumigatus* with animals). All animals used in this study were housed in groups of five in individually ventilated cages and were cared for in strict accordance to the principles outlined in the by the Brazilian College of Animal Experimentation (Princípios Éticos na Experimentação Animal - Colégio Brasileiro de Experimentação Animal, COBEA) and Guiding Principles for Research Involving Animals and Human Beings, American Physiological Society. All efforts were made to minimize suffering. Animals were clinically monitored at least twice daily by a veterinarian and humanely sacrificed if moribund (defined by lethargy, dyspnoea, hypothermia and weight loss).

### Strains and culture conditions

The *A. fumigatus* strains used in this study are CEA17 (*pyrG^−^*), Af293 (wild-type), *ΔcalA* and *ΔcrzA* (Soriani *et al.*, 2008), CEA17-80 (as the wild-type in all the experiments), *ΔpmcA* (*ΔpmcA::pyrG*), Δ*pmcB* (*ΔpmcB::pyrG*), *ΔvcxA* (Δ*vcxA::pyrG*). *ΔpmcA::pmcA* ^+^ and *alcA::pmcC*. The media used were of two basic types, i.e. complete and minimal. The complete media comprised the following three variants: YAG (2% w/v glucose, 0.5% w/v yeast extract, 2% w/v agar, trace elements), YUU [YAG supplemented with 1.2 g l-1 (each) of uracil and uridine], and liquid YG or YG + UU medium with the same composition (but without agar). A modified minimal medium (MM: 1% glucose, original high nitrate salts, trace elements, 2% agar, pH 6.5) was also used. Expression of *pmcC* gene, under the control of *alcA* promoter, was regulated by carbon source: repression on glucose 4% w/v, derepression on glycerol, and induction on threonine. Therefore, MM + Glycerol and MM + Threonine were identical to MM, except that glycerol (2% v/v) and/or threonine (100 mM) were added, respectively, in place of glucose as the sole carbon source. Trace elements, vitamins, and nitrate salts were included as described by [43]. Strains were grown at 37°C unless indicated otherwise. Additionally, 10% fetal bovine serum (Gibco) was used as a medium.

### Construction of the *A. fumigatus* mutants

A gene replacement cassette was constructed by “*in vivo*” recombination in *S. cerevisiae* as previously described [44]. Briefly, approximately 2.0 kb regions on either side of each ORF were selected for primer design. For construction, the primers were named as 5F and 5R, were used to amplify the 5′-UTR flanking region of the targeted ORF. Likewise, the primers 3F and 3R were used to amplify the 3′-UTR ORF flanking region, and the primers 5F and 3R also contains a short homologue sequence to the MCS of the plasmid pRS426. Both fragments, 5- and 3-UTR, were PCR-amplified from *A. fumigatus* genomic DNA (gDNA). The *pyrG* used in the *A. fumigatus* cassette for generating the mutant strains were used as marker for prototrophy. Deletion cassette generation was achieved by transforming each fragment along with the plasmid pRS426 *BamHI*/*EcoRI* cut in the in *S. cerevisiae* strain SC94721 by the lithium acetate method [45]. The DNA of the yeast transformants was extracted by the method described by Goldman *et al.* [46], dialysed and transformed by electroporation in *Escherichia coli* strain DH10B to rescue the pRS426 plasmid harboring the cassette. The cassette was PCR-amplified from these plasmids and used for *A. fumigatus* transformation. Southern blot analyses were used throughout of the manuscript to demonstrate that the transformation cassettes had integrated homologously at the targeted *A. fumigatus* loci. For the construction of the *alcA::pmcC* strain, 1000 bp of the *pmcC* encoding region was cloned downstream to the *alcA* promoter into the pMCB17apx vector. This construction was further transformed in *A. fumigatus* to replace the endogenous *pmcC* promoter yielding the strain *alcA::pmcC.* The *ΔpmcA* mutant strain was complemented by co-transforming a *pmcA* ^+^ DNA fragment (approximately 1 kb from each 5′ and 3′-flanking regions plus the ORF) together with the pHATα vector [47] and selecting for hygromycin resistance in MM plates with 150 µg/ml of hygromycin B.

### RNA extraction and real-time PCR reactions

After treatment conditions, mycelia were harvested by filtration, washed twice with H_2_O and immediately frozen in liquid nitrogen. For total RNA isolation, the germlings were disrupted by grinding in liquid nitrogen with pestle and mortar. Total RNA was extracted with Trizol reagent (Invitrogen, USA). Ten micrograms of RNA from each treatment was then fractionated in 2.2 M formaldehyde, 1.2% w/v agarose gel, stained with ethidium bromide, and then visualized under UV light. The presence of intact 25S and 18S ribosomal RNA bands was used to assess the integrity of the RNA. RNasefree DNase I treatment, for the real-time PCR experiments, was carried out as previously described [48]. Twenty micrograms of total RNA was treated with DNase, purified using a RNAeasy kit (Qiagen) and cDNA was generated using the SuperScript III First Strand Synthesis system (Invitrogen) with oligo(dT) primers, according to the manufacturer's protocol.

All the PCR reactions were performed using an ABI 7500 Fast Real-Time PCR System (Applied Biosystems, USA) and Taq-Man Universal PCR Master Mix kit (Applied Biosystems, USA). The reactions and calculations were performed according to Semighini *et al*. [48]. The primers and Lux™ fluorescent probes (Invitrogen, USA) used in this work are described in Supplementary Table S1.

### Cloning the crzA gene into the pDEST15 vector

The Gateway Technology (Invitrogen) was used to construct, in *Escherichia coli*, the expression system consisting of the *CRZ*A gene N-tagged to the GST gene. Briefly, the coding region of the exon 2 from CrzA was amplified from the cDNA sample by PCR using Platinum® Taq DNA Polymerase High Fidelity. (Invitrogen) and specific primers (CRZ-exon2-attB1-F 5′- GGGGACAAGTTGTACAAAAAAGCAGGCTTCGAAGGAGATAGAACCATGTCCCGCGGGCGTAGCAAG-3′ and CRZ-attB2-R 5′- GGGGACCACTTTGTACAAGAAAGCTGGGTCTCAATAGAAGTTACCGGCAGCAG-3′). Amplification was run for 30 cycles consisting of denaturation at 94°C for 1 min, primer annealing at 55°C for 1 min and primer extension at 68°C for 2 min. The PCR product carrying the attB sites was purified from agarose gel using the QIAquick PCR purification kit (Qiagen) and cloned into the pDONR201 plasmid (Invitrogen) using the BP Clonase. The BP clonase catalyze the *in vitro* recombination of PCR products or DNA segments from clones (containing attB sites) and a donor vector (containing attP sites) to generate entry clones. The entry clone pDONR201-CrzA was transformed into *E. coli* DH10B competent cells and selected for kanamicyn resistence. Entry clones were checked by sequencing using the ATT primers (ATT1F- 5′- TCGCGTTAACGCTAGCATGGATCTC-3′ and ATT2 R- 5′- GTAACATCAGAGATTTTGAGACAC-3′) and further used in LR reactions (Invitrogen) with the vector pDEST15 (an N-terminal GST fusion vector containing the T7 promoter) to generate the expression vector pDEST15-GST/CrzA.

### Production and Purification of GST::CrzA

*A. nidulans* CrzA was expressed as a GST-fusion protein from the construct pDEST15-GST/CrzA in *E. coli* Rosetta™ (DE3) pLysS strain (Novagen). Cells harboring the plasmid construction were grown in 1 L of LB medium to an O.D._600 nm_ of 0.8 and protein expression was induced at 12°C, 180 rpm overnight with 0.4 mM IPTG final concentration. After induction, cells were harvested by centrifugation, suspended in phosphate-buffered saline solution (500 mM NaCl, 2.7 mM KCl, 100 mM Na_2_HPO_4_, 2 mM KH2PO4, 5% v/v glycerol, 0.5% NP-40, pH 7.4) containing 10 mM benzamidine, 0.5 mM EDTA and 2 mM of each DTT and PMSF and lysed by sonication (ten 30 sec pulses on ice) in a Vibra-Cell disrupter (Sonics®). Cell lysate was clarified at 23,000*× g*, 20 min, 4°C, and the recombinant protein was purified by affinity chromatography on a GSTrap FF column (GE HealthCare) according to manufacturer's instructions on an ÄKTA Prime purification system. Recombinant protein was eluted in a linear gradient of 20 mM glutathione in 50 mM Tris-HCl, 500 mM NaCl, 5% v/v glycerol, 2 mM DTT, pH 8.0 buffer. Chromatographic fractions were analyzed by SDS-PAGE followed by Coomassie Brilliant Blue staining [49] and fractions containing the purified protein were combined, concentrated and quantified using BSA as standard [50].

### Electrophoretic Mobility Shift Assay

GST*::*CrzA recombinant protein was assayed in DNA-protein binding reactions using three 300 bp DNA fragments of the *pmcA*, *pmcB* and *pmcC* promoters as probes, containing the putative *cis-*regulatory calcineurin-dependent response elements (CDREs) for the transcription factor CrzA (Supplementary Table S1 and Figure S1). Binding reactions were carried out in 1×binding buffer (25 mM HEPES- KOH, pH 7.9, 20 mM KCl, 10% w/v glycerol, 1 mM DTT, 0.2 mM EDTA, pH 8.0, 0.5 mM PMSF, 12.5 mM benzamidine, 5 mg/mL of each antipain and pepstatin A) containing 2 µg poly(dI-dC).(dI-dC) as non-specific competitor and 1–2 µg of GST*::*CrzA recombinant protein, at room temperature for 10 min. After that, DNA probes (10^4^ cpm) were added and the binding reactions were incubated at room temperature during 20 min prior to being loaded onto a native 5% polyacrylamide gel in 0.5× TBE buffer. Gels were run at 10 mA, 15°C, dried, and exposed to X-ray film. For competition assays, a molar excess of the specific DNA competitors were added prior to incubation with the radiolabeled probe.

### DNA Probes and Specific Competitors for EMSA

Putative *cis* CrzA motifs were visually identified in the promoter regions of the genes *pmcA*, *pmcB* and *pmcC* by using the *A. fumigatus* CDRE consensus. To produce the *pmcA* probe, a 300 bp DNA fragment of the *pmcA* promoter was amplified from *A. fumigatus* genomic DNA by using the primers PMCA-5R and 5′-PMCA (Supplementary Table S1) in the presence of [α-^32^P]-dATP (3,000 Ci/mmol) and purified on 2% low-melting point agarose gel. *pmcB* and *pmcC* probes were prepared as above using the primer pairs PMCB-5R and 5′-PMCB (Supplementary Table S1), and PMCC-5R and 5′-PMCC (Supplementary Table S1). The unlabeled 300 bp *pmcA*, *pmcB* and *pmcC* probes were used as specific DNA competitors which were quantified by measuring the absorbance at 260 nm and added to the binding reaction in a 30- to 50-fold molar excess, 10 min prior to the addition of the respective probes. DNA oligonucleotides containing the CDRE motifs identified in *pmcA* and *pmcC* probes were also used as specific competitors after annealing the complementary oligonucleotides pairs pmcA1/pmcA2 and pmcC1/pmcC2, respectively (Supplementary Table S1). The DNA oligonucleotides were quantified by measuring the absorbance at 260 nm and added to the reaction in 10–30 fold molar excess.

Mutated probes (m*pmcA* and m*pmcC*) were prepared by changing the element core sequences by site-directed mutagenesis in a two-step PCR. In the m*pmcA* probe the sequence 5′-CCCTGCCCC-3′ was changed to 5′-AAAGTAAAA-3′ by using the oligonucleotide pair mPMCA-F and mPMCA-R in the in the first reaction to amplify two fragments. The oligonucleotide pair PMCA-5R and 5′-PMCA (Supplementary Table S1) was used in a second reaction to amplify the whole DNA fragment containing the mutation. In the m*pmcC* probe the sequence 5′-CACAGCCAC-3′ was changed to 5′-ACACTAACA-3′ by using the oligonucleotide pair mPMCC-F and mPMCC-R in the in the first reaction. The oligonucleotide pair PMCC-5R and 5′-PMCC (Supplementary Table S1) was used in a second reaction to amplify the whole DNA fragment containing the mutation. For EMSA, both mutated fragments were used as templates in PCR amplifications in the presence of [α-^32^P]-dATP (3,000 Ci/mmol) and purified on 2% low-melting point agarose gel.

### Determination of the relative levels of intracellular calcium concentration

To investigate the relative intracellular free calcium concentration we used the Fura-2 acetoxymethyl ester (Fura-2-AM; Invitrogen). Briefly, 10^7^ conidia of each wild-type, *ΔpmcA*, *ΔpmcA::pmcA^+^*, *ΔpmcB*, and *ΔpmcB::pmcB^+^* were incubated in YG medium for 8 hours with shaking at 37°C. Then, each strain was either treated with 500 mM CaCl_2_, or not, in fresh YG medium for 30 minutes. After incubation the cells were washed three times with PBS and loaded with 10 µM Fura-2-AM for 30 min at 37°C. After washing, Fura-2 fluorescence was measured by alternating the excitation wavelengths at 340 and 380 nm with an emission wavelength fixed at 505 nm. The relative intracellular calcium concentration is expressed as the ratio between fluorescence intensities with excitation wavelengths at 340 and 380 nm. All data presented are representative of three independent experiments.

### Murine model of pulmonary aspergillosis

Outbred female mice (BALB/c strain, 20–22 g) were housed in individually vented cages, containing 5 animals. Mice were immunosuppressed with cyclophosphamide at 150 mg/kg of body weight, administered intraperitoneally on days −4, −1 and 2, and hydrocortisonacetate was injected subcutaneously at 200 mg/kg on day −3, modified from [51]. *A. fumigatus* spores for inoculation were grown on *Aspergillus* complete medium for 2 days prior to infection. Conidia were freshly harvested using sterile PBS and filtered through Miracloth (Calbiochem). Conidial suspensions were spun for 5 min at 3000 *g*, washed three times with sterile PBS, counted using a hemocytometer and re-suspended at a concentration of 2.5×10^6^ conidia/ml. Viable counts from administered inocula were determined following serial dilution by plating on *Aspergillus* complete medium and grown at 37°C. Mice were anaesthetized by halothane inhalation and infected by intranasal instillation of 5.0×10^4^ conidia in 20 µl of PBS. As negative control, a group of 5 mice received only PBS intranasally. Mice were weighed every 24 h from the day of infection and visually inspected twice daily. In the majority of cases the end-point for survival experimentation was when a 20% reduction in body weight measured from the day of infection and at this point the mice were sacrificed. Significance of comparative survival was calculated using Log Rank analysis in the Prism statistical analysis package. Additionally, at 3 days post infection, 2 mice per strain were sacrificed, from which the lungs were removed, fixed and processed for histological analysis.

### Lung histopathology and fungal burden

After sacrifice, the lungs were removed and fixed for 24 h in 10% buffered formalin phosphate. Samples were washed in 70% alcohol several times, dehydrated in alcohols of increasing concentrations, diafanized in xylol and embedded in paraffin. For each sample, sequential 5 µm sections were collected on glass slides and the sections were stained with Gomori methenamine silver (GMS) or hematoxylin and eosin (HE) stain following standard protocols [24]. Briefly, sections were deparaffinized, oxidized with 4% chromic acid, stained with methenamine silver solution, and counter stained with picric acid or light green. For HE staining, sections were deparaffinized, stained first with hematoxylin and then stained with eosin. All stained slides were immediately washed, preserved with mounting medium and sealed with a cover glass. Microscopical analyses were done using an Axioplan 2 imaging microscope (Zeiss) at the stated magnifications under brightfield conditions.

To investigate fungal burden in murine lungs, mice were immunosuppressed with cyclophosphamide at 150 mg/kg of body weight administred intraperitoneally on days −4 and −1 and hydrocortisonacetat injected subcutaneously at 200 mg/kg on day −3. Five mice per group (wild-type, *ΔpmcA*, *ΔpmcA::pmcA*, and PBS control) were inoculated with 5×10^5^ conidia/20 µl suspension intranasally. A higher inoculum, in comparison to the survival experiments, was used to increase fungal DNA detection. Animals were sacrificed 48 hours post infection, both lungs were harvested and immediately frozen in liquid nitrogen. A mortar and pestle were used to pulverize the samples (frozen in liquid nitrogen) and DNA was extracted by the Phenol/Chlroform method. DNA quantity and quality was assessed with a NanoDrop 2000 (Thermo Scientific). Around 200 ng of total DNA of each sample was used for quantitative Real-Time PCR reaction. A primer and a Lux™ probe (invitrogen) were used to amplify the 18S rRNA region of *A. fumigatus* (primer: 5′-CTTAAATAGCCCGGTCCGCATT-3′, probe: 5′-CATCACAGACCTGT TATTGCCG-3′) and an intronic region of mouse GAPDH (primer: 5′-CGAGGGACTTGGAGGACACAG-3′, probe: 5′-GGGCAAGGCTAAAGGTCAGCG-3′). Six-point standard curves were calculated using serial dilutions of gDNA from all *A. fumigatus* strains used here and non-infected mouse lung. Fungal and mouse DNA quantities were obtained from the Ct values from an appropriate standard curve. Fungal burden was determined via the ratio between ng of fungal and mouse DNA.

### Bronchoalveolar lavages

To analyze gene expression of *A. fumigatus* strains during early pulmonary infection, Outbred female mice (BALB/c strain, 20–22 g) were housed in individually vented cages, containing 5 animals. Mice were immunosuppressed with cyclophosphamide (Genuxal, Baxter) at 150 mg/kg of body weight administered intraperitoneally on days −4 and −1 and hydrocortisone sodium succinate (Hidrosone, Cellofarm) was injected subcutaneously at 200 mg/kg on day −1. All mice received tetracycline hydrochloride 0.5 mg/L in drinking water, as prophylaxis against bacterial infection. *A. fumigatus* spores for inoculation were grown on *Aspergillus* complete solid medium (YAG) for 2 days prior to infection. Conidia were freshly harvested using sterile PBS and filtered through Miracloth (Calbiochem). Conidial suspensions were spun for 5 min at 4,000 rpm, washed three times with sterile PBS, counted using a hemocytometer and re-suspended at a concentration of 2.5×10^10^ conidia/ml. Five mice per group (wild-type, *ΔcrzA* and *ΔcalA*) were anesthetized by isoflurane (Isothane, Baxter) inhalation and infected by intranasal instillation of 10^9^ conidia in 40 µl of PBS. Groups of infected mice were sacrificed and processed collectively at time points 4 and 12 hours post-infection. Bronchoalveolar lavage (BAL) was performed immediately after culling using three 0.5 ml aliquots of cold sterile PBS. To remove the mice cells from BALs, samples were spun down in microcentrifuge tubes, supernatants were removed, the samples were resuspended in 1 ml of sterile ultrapure water, centrifuged again and finally the pellets were snap frozen immediately using liquid nitrogen. To extract RNA, BAL samples from each strain (5 BALs per strain) were mixed with 1 ml Trizol LS Reagent (Invitrogen) and acid treated glass beads (425–600 µm, Sigma-Aldrich). Fungal cells were homogenized by 10 min vortexing, centrifuged at 12,000 rpm for 10 min, the upper phase was mixed with 200 µl chloroform, centrifuged again, the new upper phase was mixed with 500 µl isopropanol and incubated overnight at −80°C. After washing the pellet with 70% ethanol, RNA was dissolved in 20 µl DEPC water. Further RNA purification was carried out using RNeasy mini kit (Qiagen), following manufacturer's instructions. RNA concentration and integrity was monitored by NanoDrop® 2000 – Thermo Scientific (Uniscience). RNA amplification was done according to Agilent Low RNA Input Fluorescent Linear Amplification kit (Agilent Technologies). RNAse free DNAse treatment was carried out as previously described [48].

## Supporting Information

Figure S1 (A) Clustal alignment of *A. fumigatus* PmcA, PmcB, and PmcC.
(DOCX)

Figure S2 (B) CrzA-binding regions of the *pmcA-C* genes (upstream the ATG start codon). Underline and in bold the putative CDRE-motif.
(DOCX)

Figure S3 Southern blot and PCR analyses for (A) *ΔvcxA*, (B) *ΔpmcB*, (C) *ΔpmcA* and (D) *alcA::pmcC.*
(PPT)

Figure S4 Histological analysis of alveolar lavages after infection with the *A. fumigatus* wild type, *ΔcalA*, and *ΔcrzA* strains. Germlings and host cells were detected by using Grocotts methenamine silver and haematoxylin and eosin staining, respectively. Bars, 100 µm.
(PPTX)

Figure S5 *A. fumigatus ΔpmcB* virulence studies in neutropenic mice. Comparative analysis of wild type and *ΔpmcB* strains in a neutropenic murine model of pulmonary aspergillosis. A group of 10 mice per strain was infected intranasally with 20 µl suspension of conidiospores at a dose of 2.0–5.0×10^4^.
(PPTX)

Figure S6 (A) Growth phenotype of the *ΔpmcA::pmcA^+^* strain grown on YAG+CaCl_2_ 500 mM. (B) PCR of the *pmcA* open reading frame. (C) PCR of the *pmcB* open reading frame.
(PPT)

Table S1 List of primers and probes used in this work.
(DOC)
