# Correction: Functional Characterization of an *Aspergillus fumigatus* Calcium Transporter (PmcA) that Is Essential for Fungal Infection

**DOI:** 10.1371/annotation/bf626b67-ad61-4050-8cc9-5016aa8ab6b8

**Published:** 2012-11-20

**Authors:** Taísa Magnani Dinamarco, Fernanda Zanolli Freitas, Ricardo S. Almeida, Neil Andrew Brown, Thaila Fernanda dos Reis, Leandra Naira Zambelli Ramalho, Marcela Savoldi, Maria Helena S. Goldman, Maria Célia Bertolini, Gustavo Henrique Goldman

Supplementary Figure S4 (Histological analysis of alveolar lavages after infection with the A. fumigatus wild-type, delta cnaA, and delta crzA) is incorrect. The experiments in the article were performed with BALB/c strain. However, the experiments shown in Figure S4 were performed with CD1 animals. This should be removed from the Supporting Information section of the manuscript. Accordingly, any reference to this Figure in the text should be replaced by "data not shown". 

