# Supplementary material for: Functional Characterization of an Aspergillus fumigatus Calcium Transporter (PmcA) that Is Essential for Fungal Infection
Source: PLoS One. 2012 May 23;7(5):e37591. doi: 10.1371/journal.pone.0037591 (PMC3359301; doi:10.1371/journal.pone.0037591)
Supplement: Figure S1 — (A) Clustal alignment of A. fumigatus PmcA, PmcB, and PmcC. (DOCX) [file pone.0037591.s001.docx]

Supplementary Figure S1 – (A) Clustal alignment of *A. fumigatus* PmcA, PmcB, and PmcC.

Afu1g10880|pmcA MSSNPNQDAKTPLRRERAPTITIDTSAVTSTPDPSQPTLQVSTEPADASD

Afu3g10690|pmcB MSKRQSLSSQPSEQQ----ALSLDI---PSTVDSNRLTVQ----------

Afu7g01030 MSLRLVSSVRDDLFA----AVASDT-------------------------

** . . : ::: *

Afu1g10880|pmcA TSALLYNGSPSPTDLRSSASIRSSASSEGRDHESRPTSPHNVSSPTSKLA

Afu3g10690|pmcB -----------------------------ND---HPRTPNSIDGDTLRS-

Afu7g01030 --------------------------------------------------

Afu1g10880|pmcA ESMSNSNFLSVPGT--RSRGNSLESEDSSQSTSTYGGETYVGSPSQGARP

Afu3g10690|pmcB ---RSESFASNADTIARSRANS---------EFTISKEAYDDVP------

Afu7g01030 --------------------------------------------------

Afu1g10880|pmcA EHGNKNGTNGKILSDEEALKPDPGREAEFQVEDNKFAFSPGQLNKLLNPK

Afu3g10690|pmcB --------------LSEALTPDPRNEQDFRVENNKFAFSPGQLNKMLNPK

Afu7g01030 ----------------------------QAAQDNCFAFSPDQLNQLFNPK

.::* *****.***:::***

Afu1g10880|pmcA SLGAFHALGGLQGLERGLRTNLRSGLSVDETTLEGTVSFEEVASSGAQNT

Afu3g10690|pmcB SLAAFQALGGLRGLEKGLRTDLTSGLSEDEALLDGTVDFQEAT----QND

Afu7g01030 SPPALYALGGLYGLEYGLRTDLSAGLSANERILPGAVTLEEAR----QAA

* *: ***** *** ****:* :*** :* * *:* ::*. *

Afu1g10880|pmcA LPKSDSDPPNQGSPARSNTAPARRHDDAFSDRKRIYGLNKLPEKKPKSIL

Afu3g10690|pmcB QK--LSKQISHNAPVAPAPAPELGGGSRFQDRIRVFSQNKLPARKSTGFL

Afu7g01030 LCQTESKRPLLANAAR--PHPNQEPSVPFSDRTRVFGRNVLPDAKRKGFG

*. .. . * . *.** *::. * ** * ..:

Afu1g10880|pmcA ELAWIAYNDKVLILLTIAAVISLALGIYQSVTATDGEARVQWVEGVAIIV

Afu3g10690|pmcB KLLWFAYNDKIIILLTIAAIVSLSLGIYETVD---EGHGVDWIEGVAICV

Afu7g01030 RLLWDAYNDKIIILLTIAAVVSLALGIYEAVS---GQSQVDWIEGVAVCV

.* * *****::*******::**:****::* *:*:****: *

Afu1g10880|pmcA AIVIVVVVGAANDWQKERQFVKLNKKKEDRQVKVIRSGKTVEISIHDVLV

Afu3g10690|pmcB AILIVTVVTAVNDLQKERQFAKLNKRNSDREVKAVRSGKVAMISVFDITV

Afu7g01030 AIVIVVAATAGNDWQKERQFARLNQLKADRQVRVIRSGRPMMLHINDLVV

**:**... * ** ******.:**: : **:*:.:***: : : *: *

Afu1g10880|pmcA GDVMHLEPGDLVPVDGVFITGHNVKCDESSATGESDVLRKTPGSDVYQAI

Afu3g10690|pmcB GDVLHLEPGDSVPADGILISGHGIKCDESSATGESDAMKKTNGHEVWQRI

Afu7g01030 GDVVHVGPGDCAPADGVVITSHGLKCDESLATGESDQVEKVS--------

***:*: *** .*.**:.*:.*.:***** ****** :.*.

Afu1g10880|pmcA ERHENLKKLDPFIVSGAKVSEGVGTFLVTAVGVNSTYGKTLMSLQDEGQT

Afu3g10690|pmcB VNGTATKKLDPFMISGSKVLEGVGTYLVTSVGPYSSYGRILLSLQESNDP

Afu7g01030 -AGAATDDQDPFIISGSKVLEGMGTYLVTSVGPHSTYGRIMVSLGTESAP

.. ***::**:** **:**:***:** *:**: ::** .. .

Afu1g10880|pmcA TPLQSKLNVLAEYIAKLGLAAGLLLFIVLFIKFLAQLKDMYGA-DAKGQA

Afu3g10690|pmcB TPLQVKLGRLANWIGWLGSSAAIILFFALFFRFVAQLPNNPASPAVKGKE

Afu7g01030 TPLQVKLGKLANWIGWFGLGAALLLFFVLLFRFLAQLPDNDAPSTVKGQE

**** **. **::*. :* .*.::**:.*:::*:*** : .. .**:

Afu1g10880|pmcA FLQIFIVAVTIIVVAVPEGLPLAVTLALAFATTRMLKDNNLVRLLRACET

Afu3g10690|pmcB FVDILIVAVTVIVVAIPEGLPLAVTLALAFATTRMVKENNLVRVLRACET

Afu7g01030 FMDILIVTVTVIVVAIPEGLPLAVTLALAFATARMLKENNLVRQLRACET

*::*:**:**:****:****************:**:*:***** ******

Afu1g10880|pmcA MGNATTICSDKTGTLTENKMTAVAATLGTSTKFGEKSAGASSGQANGVHD

Afu3g10690|pmcB MGNATVVCSDKTGTLTQNKMTVVAGTFGAQESFGQDRKE----------D

Afu7g01030 MGNATVICSDKTGTLTQNRMTVVAGFLSPSESFGQLPLE----------T

*****.:*********:*:**.**. :... .**:

Afu1g10880|pmcA ATNSSGSMSPSEFASSLASPVKALLLDSIVINSTAFEGEQDGTMTFIGSK

Afu3g10690|pmcB AEPPSDSTTVAEIFKQCSTAVRDLIIKSIALNSTAFEEEKEGSREFVGSK

Afu7g01030 ASQPQ-HDDISGVTQRYPAALKALLVKSLVVNSTAFEELRENETVLVGNN

* .. : . . .:.:: *::.*:.:****** ::. ::*.:

Afu1g10880|pmcA TETALLSFARTYLGMGSISEARSNAEIAQMVPFDSGRKCMAVVIRLENGK

Afu3g10690|pmcB TEVAMLQMARDYLGM-DVTTERGSAEIVQLIPFDSARKCMGVVNREPTAG

Afu7g01030 TEIALLRFAQTALDVRDASTERERTEIEQVYPFDSARKAMAVVYRLGT-G

** *:* :*: *.: . : * :** *: ****.**.*.** * .

Afu1g10880|pmcA YRMLVKGASEILLSKSTRII-RDPTKE--VS-DTSLSEKDRSALENIITH

Afu3g10690|pmcB YRLLVKGAAEIMVGACSSKV-SDLSASSDGVMVDLFTETDRQKMLDTIES

Afu7g01030 HRLLVKGAAEVVLGACTESTLPGLSDETSLAR-AQMSCEDRRTIHDQIDI

:*:*****:*:::. .: . : . :: ** : : *

Afu1g10880|pmcA YATQSLRTIGLVYRDFDQWPPRGAPTSEEDRSLAQFDPLFKDMVLFGIFG

Afu3g10690|pmcB YAMKSLRTIGLVYRDFPSWPPKDAHRVEDDPSAAKFEDVFRDMTWLGVVG

Afu7g01030 FSRASLRTIAIAYRELPAWNSEQAG--DNAKVSPGFDALFNNLTWIGAFG

:: *****.:.**:: * .. * :: . *: :*.::. :* .*

Afu1g10880|pmcA IQDPLRPGVTESVRQCQKAGVFVRMVTGDNIMTAKAIAQECGIFTPGGIA

Afu3g10690|pmcB IQDPLRPEVPVAIQKCRIAGVQVKMVTGDNLATATAIAQSCGIKTEDGIV

Afu7g01030 IHDPLRPEVPEAIRTCHTAGVQVKMVTGDNIHTALSIAISCGIKTEDGIA

*:***** *. ::: *: *** *:******: ** :** .*** * .**.

Afu1g10880|pmcA IEGPKFRQLSNRQMRQIIPRLQVLARSSPDDKKILVTQLRKLGETVAVTG

Afu3g10690|pmcB MEGPKFRQLSDQEMDEVIPRLQVLARSSPEDKRILVARLKKLGETVAVTG

Afu7g01030 MEGPDLRQLTEAQLKTIIPRLQVLARSSPSDKQLLVEHLKQLGETVAVTG

:***.:***:: :: :************.**::** :*::*********

Afu1g10880|pmcA DGTNDAQALKTADVGFSMGITGTEVAKEASDIILMDDNFASIVKAMAWGR

Afu3g10690|pmcB DGTNDGPALKTADVGFSMGIAGTEVAKEASSIILLDDNFKSIVTAIAWGR

Afu7g01030 DGTNDGPALKAADVGFSMGLSGTEVAREASSIILLDDNFRSIVTAIAWGR

*****. ***:********::*****:***.***:**** ***.*:****

Afu1g10880|pmcA TVNDAVKKFLQFQITVNITAVLLTFISAVASGDEESVLTAVQLLWVNLIM

Afu3g10690|pmcB AVNDAVSKFLQFQITVNITAVILTFVSSLYRSDNTSVLSAVQLLWVNLIM

Afu7g01030 CVNDAVAKFLQFQITVNITAVCLTVVTAIYSSSNESVFKAVQLLWLNLIM

***** ************** **.:::: ..: **:.******:****

Afu1g10880|pmcA DTFAALALATDPPTPHILDRRPEPRSAPLINLTMWKMIIGQSIFQLVVTL

Afu3g10690|pmcB DTFAALALATDPPTEQILHRKPVPKSASLFTVTMWKMIIGQAIYQLAVTF

Afu7g01030 DTFAALALATDPPTADILQRPPRPRSAPLFTVTMWKLMLGQSIYKLALCF

************** .**.* * *:**.*:.:****:::**:*::*.: :

Afu1g10880|pmcA VLNFAGKSIFKLS--SEDDMERLKTTVFNTFVWMQIFNQWNSRRIDNSLN

Afu3g10690|pmcB MLYFAGDKLLGSRLGTDKRQLKLDTIVFNTFVWMQIFNEFNNRRLDNKLN

Afu7g01030 TLYFAGNRILQYHTDGHQQQAELDTVIFNTFVWMQIFNELNCRRLDNKFN

* ***. :: .. .*.* :***********: * **:**.:*

Afu1g10880|pmcA IFEGIFRNRWFIGIQFIIVGGQVLIIFVGGQAFSIKPLVGYQWGVSLILG

Afu3g10690|pmcB IFEGMFRNYWFLGINCIMVGGQVMIIYVGGAAFNVTRLDAVQWGICIVCA

Afu7g01030 IFEGILRNRWFMVINALMVGGQVLIIFVGGAAFGVTRLDGPQWATCIGCA

****::** **: *: ::*****:**:*** **.:. * . **. .: .

Afu1g10880|pmcA VISLPVGVIIRLIPDEFVSRLIPRFWTRKKGPELVVSDEDRRFEWNPALE

Afu3g10690|pmcB IACLPWAVILRLTPDRPVEIIINF--------------------------

Afu7g01030 AFCIPWAAVLKLVPDRCVARLMSM--------------------------

.:* ..:::* **. * ::

Afu1g10880|pmcA EIRDQLTFLHTVRGGRLRNLKHKLQH----------PQE-----LL-PRS

Afu3g10690|pmcB --------VVLVVGTTLRPIGKAFSAISRIVSSMMWPVKRVSRRVLRRNA

Afu7g01030 --------VRTGVGVLLAPLRQMCRALVRAFHGFFH-------RVHVRDD

: * * : : :

Afu1g10880|pmcA RSGSRSREDSIPSTPVGENGGTSPQPATPESRSRKRTRSRSNSAFGPAAA

Afu3g10690|pmcB EDDSTTEKEEVPMTDV-EKQHTPKAPATPVV-------------------

Afu7g01030 EE------------------------------------------------

..

Afu1g10880|pmcA MAGVVAGSIAGWSPIERTPGENDSVGFNSNSPHGGLDNQEGIEIHPGTAA

Afu3g10690|pmcB --------------------------------------------------

Afu7g01030 --------------------------------------------------

Afu1g10880|pmcA DERLVGDYLSTSKTPPSQNPDLIPYFEHAPPARAPSSRSRRSTSGRSRSS

Afu3g10690|pmcB ----------------------------VPP-------------------

Afu7g01030 --------------------------------------------------

Afu1g10880|pmcA RSQSRQS

Afu3g10690|pmcB ITITSSX

Afu7g01030 -SNLTRR

:
