# Supplementary material for: Functional Characterization of an Aspergillus fumigatus Calcium Transporter (PmcA) that Is Essential for Fungal Infection
Source: PLoS One. 2012 May 23;7(5):e37591. doi: 10.1371/journal.pone.0037591 (PMC3359301; doi:10.1371/journal.pone.0037591)
Supplement: Figure S2 — (B) CrzA-binding regions of the pmcA-C genes (upstream the ATG start codon). Underline and in bold the putative CDRE-motif. (DOCX) [file pone.0037591.s002.docx]

Supplementary Figure S2 – CrzA-binding regions of the *pmcA-C* genes (upstream the ATG start codon). Underline and in bold the putative CDRE-motif.

*pmcA* gene

5’-GTGTGCCGCTATCTTCTTCGTGCCAGCAGGAGATTCGTCCACTCAAATCTGCATCAACTCATGAGCTTTCTCCCTGATTACGTGCCCCTTATGTATATCTGAGTTTGGTTGCGCGCCGTCTTTGATACCTGGAATGTTG**CCCTGCCCC**TTGTCACAATTTCCTTGTATGTATTAACGCGACTGCTTCATTCGCTCCTTCTCGGGCATTCCTGACCCAGAGTCTATCCCTTCCATACCACACCATTACCCAAACTCTCTCGGAATCCGCTCTGCGGGCTCCAAATCCTGGGCCATC-3’

*pmcB* gene

5’-TCTAGATAAATGATATGATACCTTCATCTTACCAAAGGCGGACTCAGCCACTGTCAAAGATCGATAAAATCCCATCCGAACCGCTTACCTCTGCTTCTGCCCGTTCTCTTACTATATTGACAGGTGATAATTGTCTTTTTCAAATCATTTTACTAATATAGCAAGCGTGTCGGTCTAATCATGCTGACACCAAACGGAAGCCGTGCATACGGCCTCCTAAGCCCTCAGGCTAATACTCAGGATAGAGAGGTATGTGATCTGCTTCATCAATAGGTATGACAGAGATATATTAACC-3

*pmcC* gene

5’- CTGCCCTGATTACCGTCATGATGATGAAGCTGATTTATAGATGATCCGGCCAAGCGAGGCAGTGCTCGGCCAAGAATCGAGAGTCGGGTTGATCCGCGTCTTCAGATTGATCGATTAGATGGGCGGATTTCCTTCTCAAGATAAAGTTATTCAATGTCCGGTGTTGATCCAAGTTACCCATGGGTGAGTCCGA**CACAGCCAC**GCTCTATGGGTTAAGGCGAAAATAATGTATATAGACAGTATATTGTCAAGACGCTCGGAGGAGGAGTGATGTCGCCAGCTTATCCACACTAAA-3’
