# Supplementary material for: Functional Characterization of an Aspergillus fumigatus Calcium Transporter (PmcA) that Is Essential for Fungal Infection
Source: PLoS One. 2012 May 23;7(5):e37591. doi: 10.1371/journal.pone.0037591 (PMC3359301; doi:10.1371/journal.pone.0037591)
Supplement: Figure S3 — Southern blot and PCR analyses for (A) ΔvcxA, (B) ΔpmcB, (C) ΔpmcA and (D) alcA::pmcC. (PPT) [file pone.0037591.s003.ppt]

## Slide 1
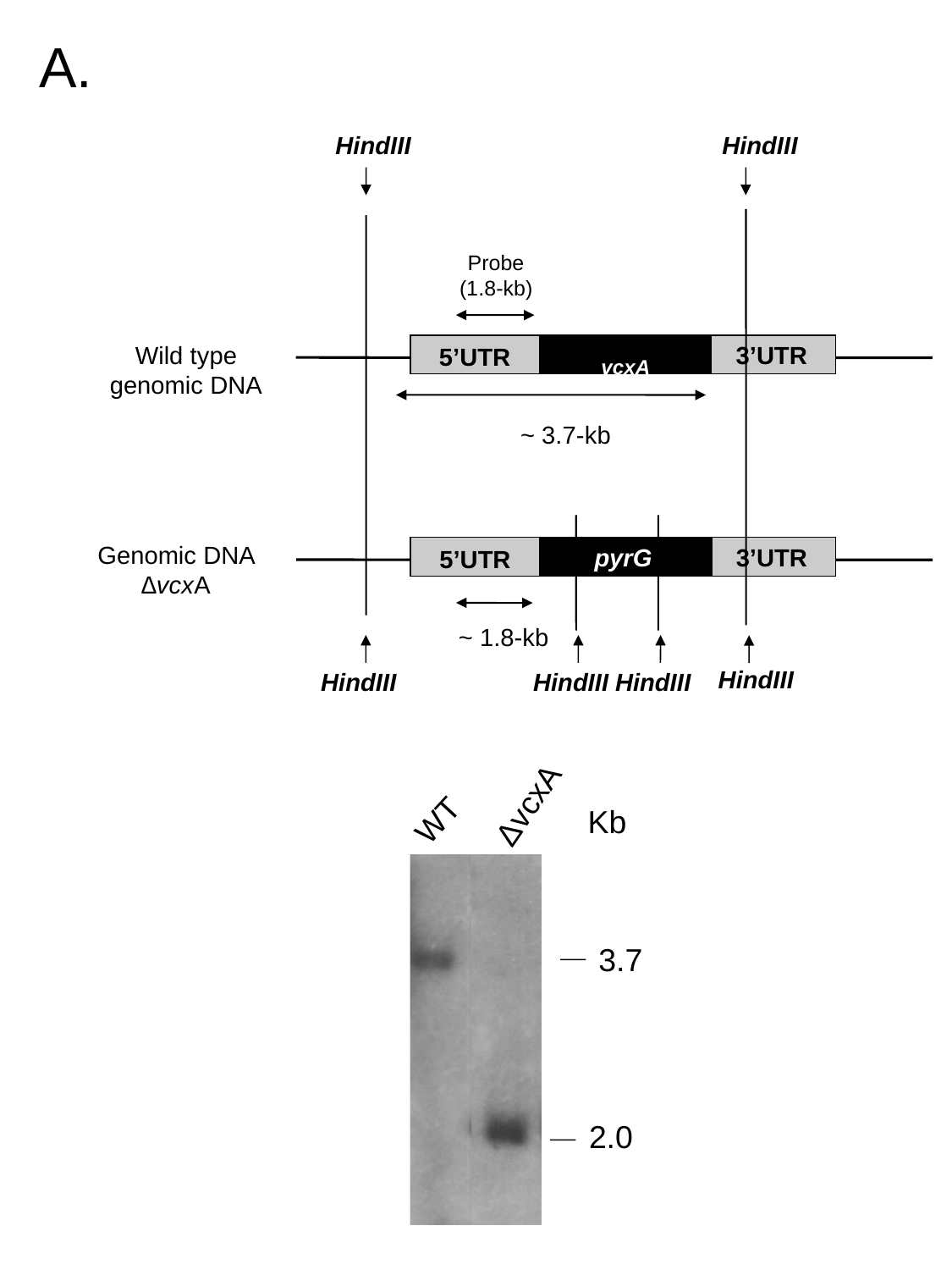

A.
HindIII
HindIII
Probe (1.8-kb)
Wild type
genomic DNA
3’UTR
5’UTR
vcxA
~ 3.7-kb
Genomic DNA ∆vcxA
3’UTR
pyrG
5’UTR
~ 1.8-kb
HindIII
HindIII
HindIII
HindIII
ΔvcxA
Kb
 WT
3.7
2.0

## Slide 2
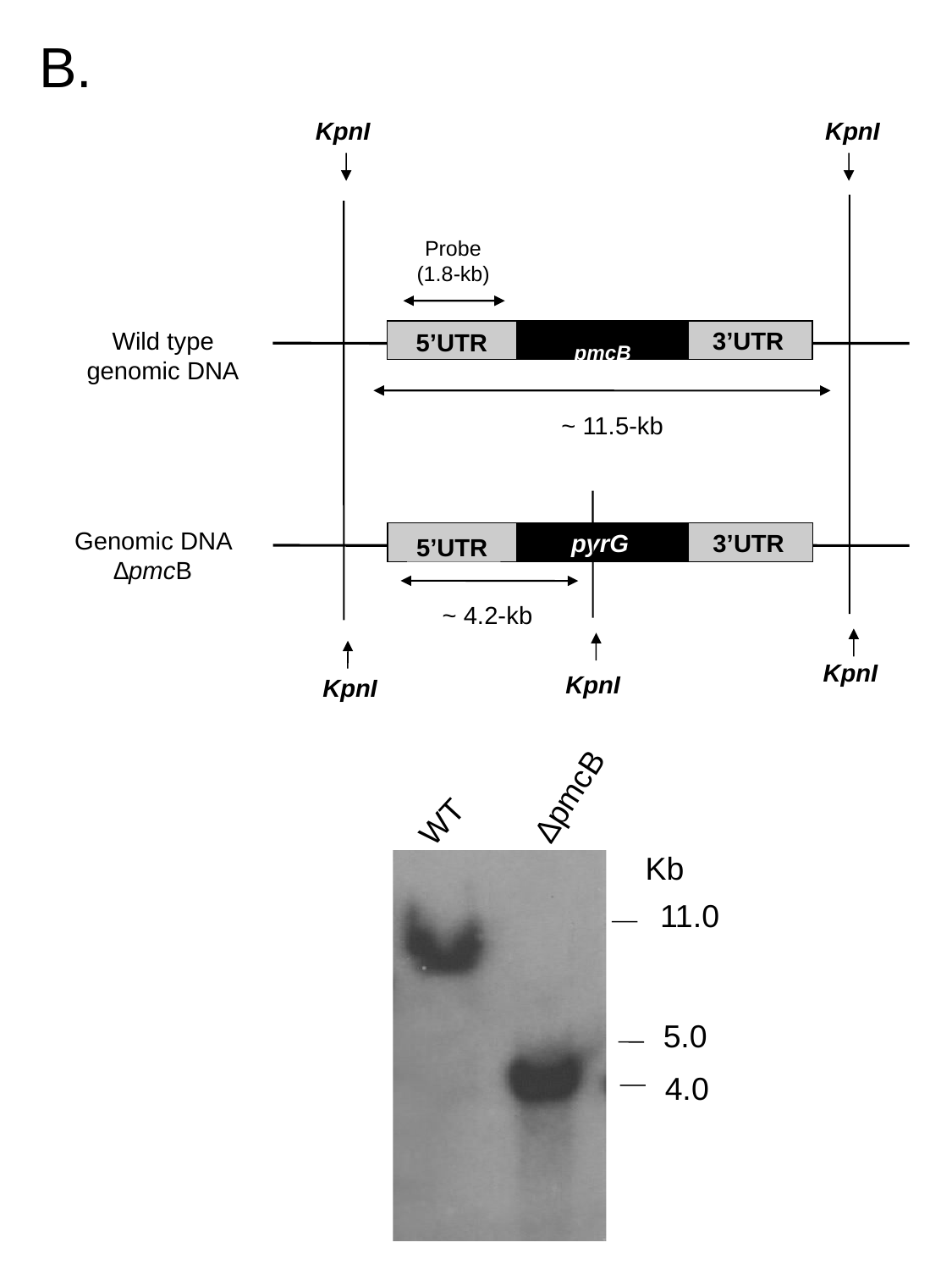

B.
KpnI
KpnI
Probe (1.8-kb)
Wild type
genomic DNA
3’UTR
5’UTR
pmcB
~ 11.5-kb
Genomic DNA ∆pmcB
3’UTR
pyrG
5’UTR
~ 4.2-kb
KpnI
KpnI
KpnI
ΔpmcB
 WT
Kb
11.0
5.0
4.0

## Slide 3
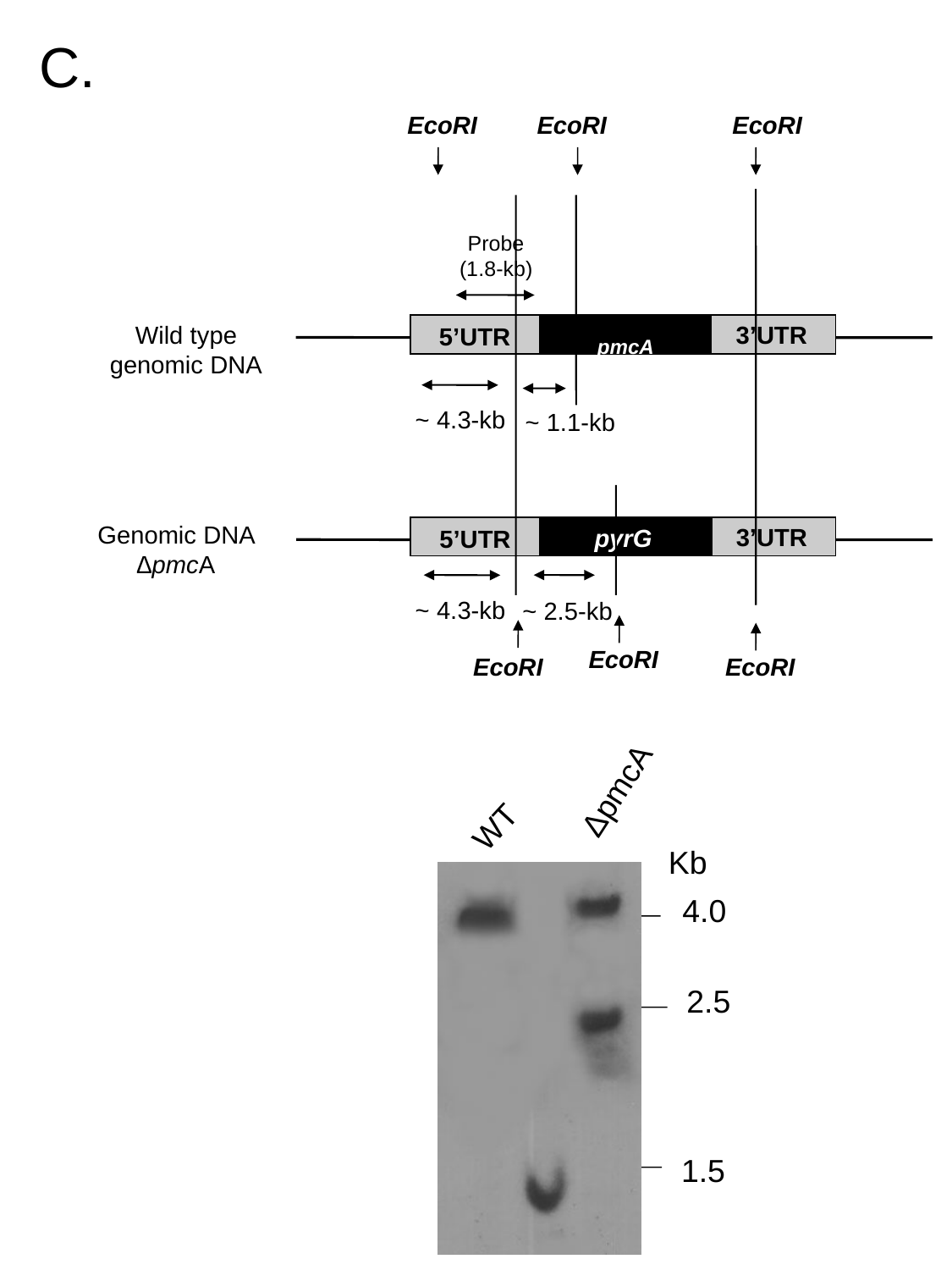

C.
EcoRI
EcoRI
EcoRI
Probe (1.8-kb)
Wild type
genomic DNA
3’UTR
5’UTR
pmcA
~ 4.3-kb
~ 1.1-kb
Genomic DNA ∆pmcA
3’UTR
pyrG
5’UTR
~ 4.3-kb
~ 2.5-kb
EcoRI
EcoRI
EcoRI
ΔpmcA
 WT
Kb
4.0
2.5
1.5

## Slide 4
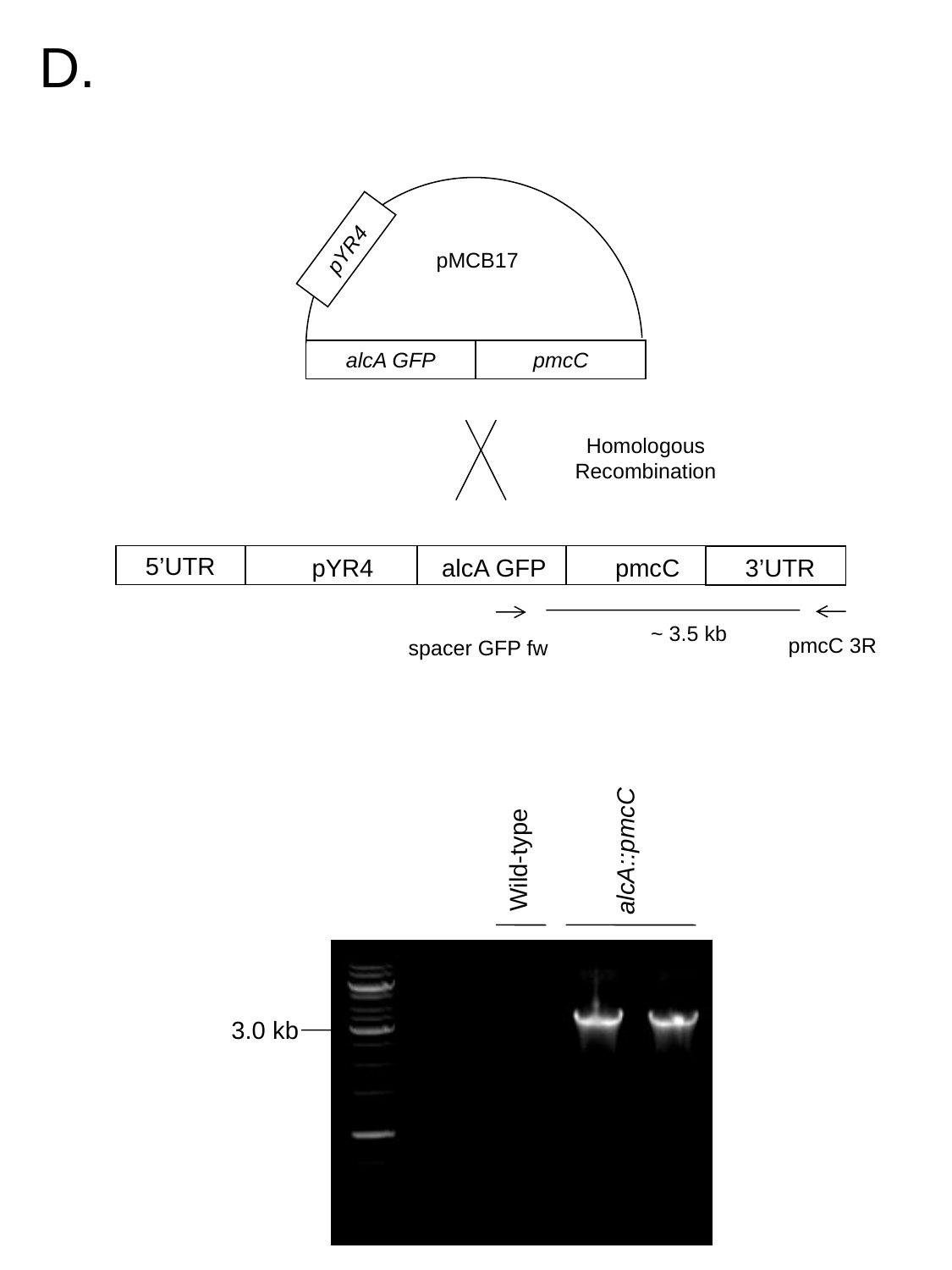

D.
pMCB17
alcA GFP
pmcC
pYR4
Homologous Recombination
5’UTR
pYR4
alcA GFP
pmcC
3’UTR
~ 3.5 kb
pmcC 3R
spacer GFP fw
alcA::pmcC
Wild-type
3.0 kb

## Slide 5
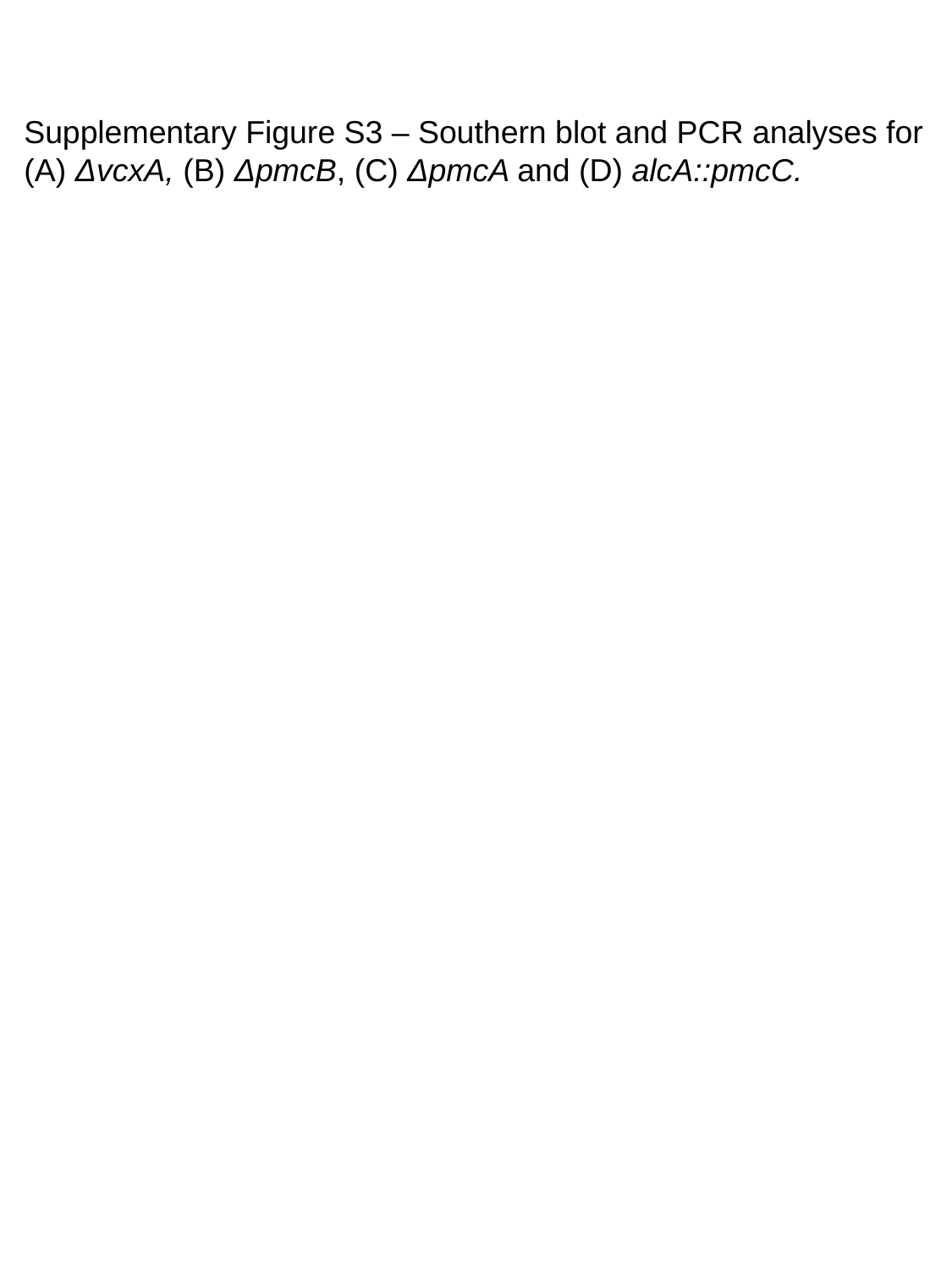

Supplementary Figure S3 – Southern blot and PCR analyses for
(A) ΔvcxA, (B) ΔpmcB, (C) ΔpmcA and (D) alcA::pmcC.
