# Supplementary material for: Functional Characterization of an Aspergillus fumigatus Calcium Transporter (PmcA) that Is Essential for Fungal Infection
Source: PLoS One. 2012 May 23;7(5):e37591. doi: 10.1371/journal.pone.0037591 (PMC3359301; doi:10.1371/journal.pone.0037591)
Supplement: Figure S4 — Histological analysis of alveolar lavages after infection with the A. fumigatus wild type, ΔcalA, and ΔcrzA strains. Germlings and host cells were detected by using Grocotts methenamine silver and haematoxylin and eosin staining, respectively. Bars, 100 µm. (PPTX) [file pone.0037591.s004.pptx]

## Slide 1
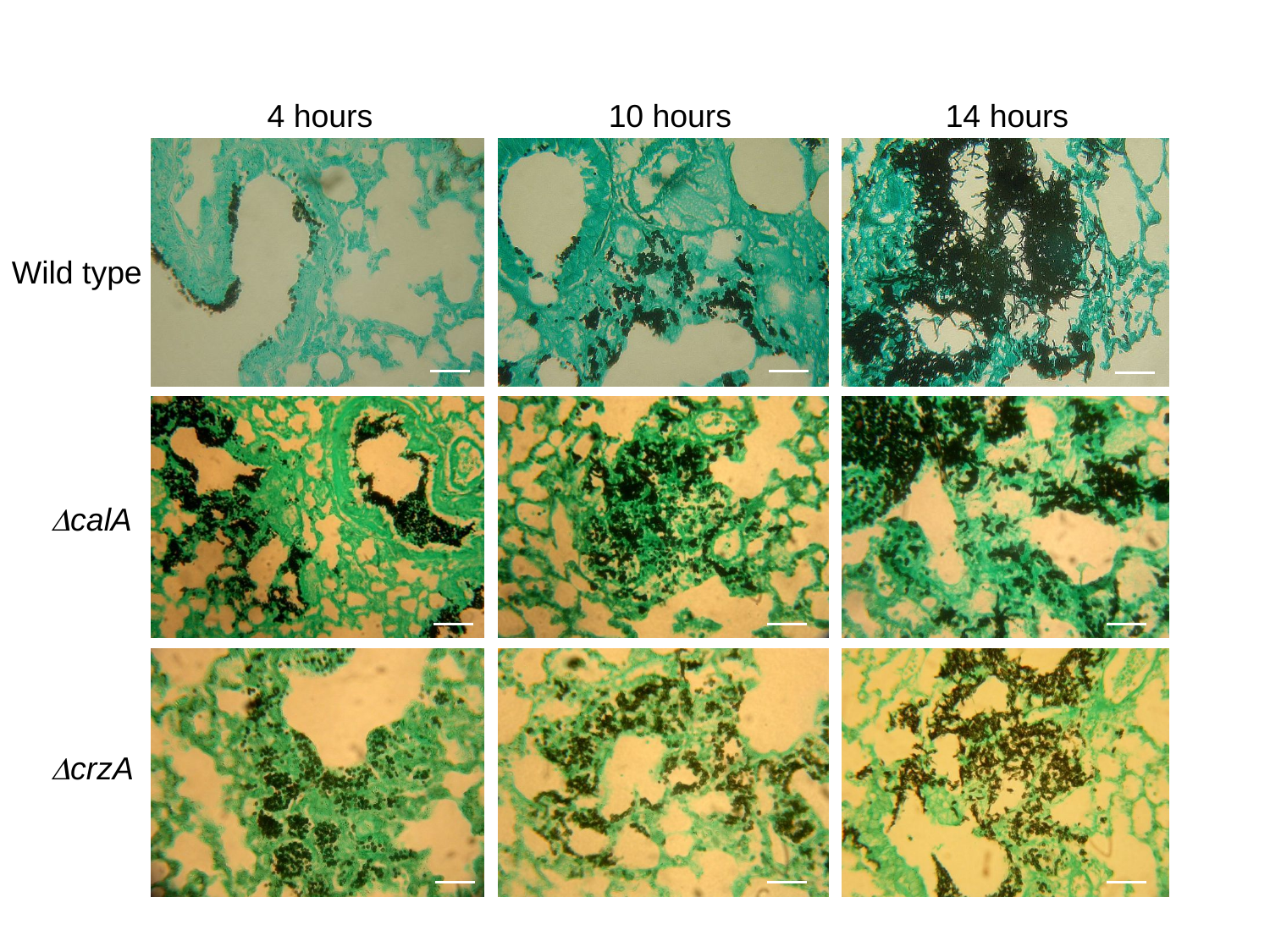

4 hours
10 hours
14 hours
Wild type
ΔcalA
ΔcrzA

## Slide 2
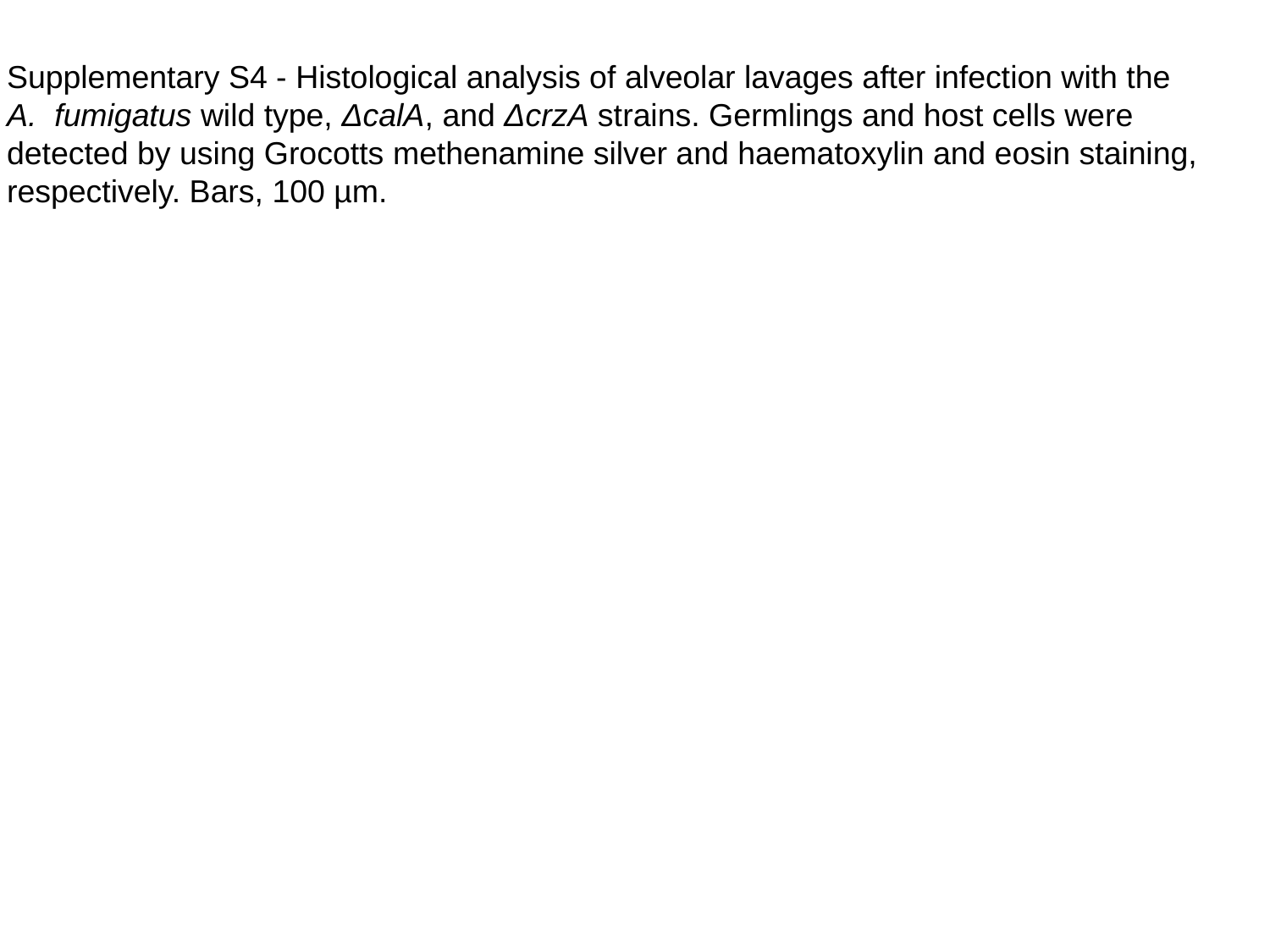

Supplementary S4 - Histological analysis of alveolar lavages after infection with the
fumigatus wild type, ΔcalA, and ΔcrzA strains. Germlings and host cells were
detected by using Grocotts methenamine silver and haematoxylin and eosin staining,
respectively. Bars, 100 µm.
