# Supplementary material for: Functional Characterization of an Aspergillus fumigatus Calcium Transporter (PmcA) that Is Essential for Fungal Infection
Source: PLoS One. 2012 May 23;7(5):e37591. doi: 10.1371/journal.pone.0037591 (PMC3359301; doi:10.1371/journal.pone.0037591)
Supplement: Figure S5 — A. fumigatus ΔpmcB virulence studies in neutropenic mice. Comparative analysis of wild type and ΔpmcB strains in a neutropenic murine model of pulmonary aspergillosis. A group of 10 mice per strain was infected intranasally with 20 µl suspension of conidiospores at a dose of 2.0–5.0×10^4^. (PPTX) [file pone.0037591.s005.pptx]

## Slide 1
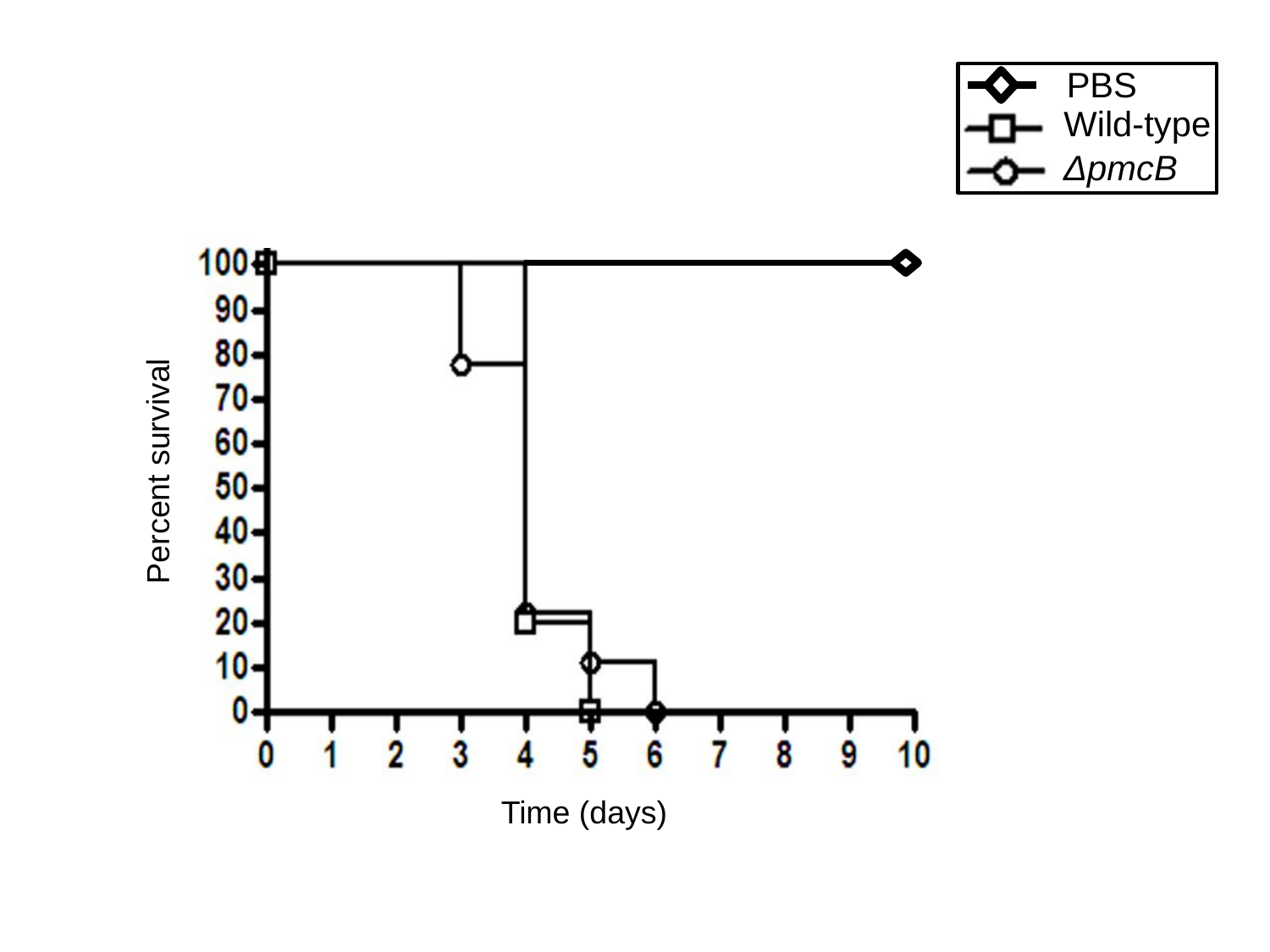

PBS
Wild-type
ΔpmcB
Percent survival
Time (days)

## Slide 2
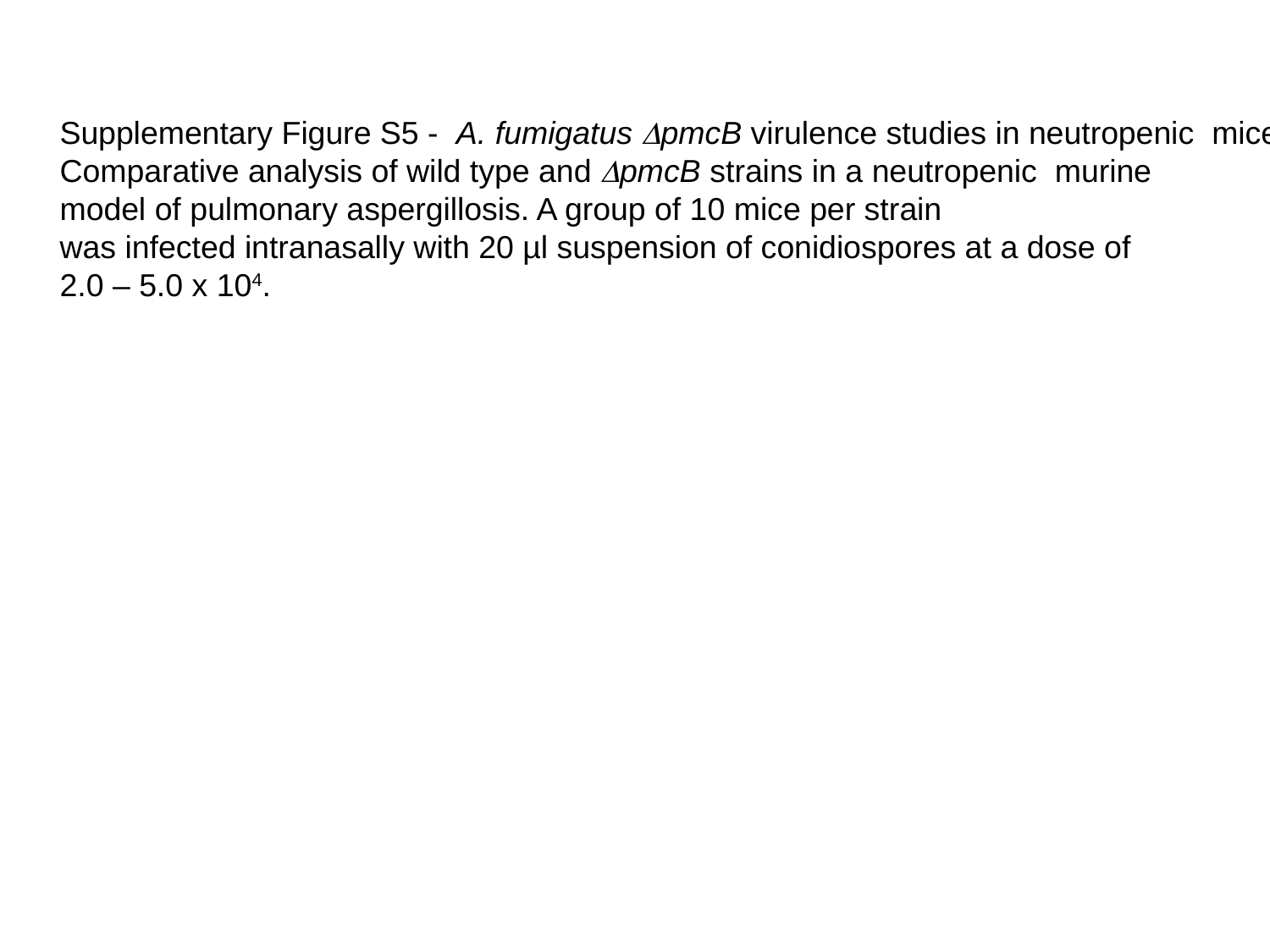

Supplementary Figure S5 - A. fumigatus ΔpmcB virulence studies in neutropenic mice.
Comparative analysis of wild type and ΔpmcB strains in a neutropenic murine
model of pulmonary aspergillosis. A group of 10 mice per strain
was infected intranasally with 20 µl suspension of conidiospores at a dose of
2.0 – 5.0 x 10^4^.
