# Supplementary material for: Functional Characterization of an Aspergillus fumigatus Calcium Transporter (PmcA) that Is Essential for Fungal Infection
Source: PLoS One. 2012 May 23;7(5):e37591. doi: 10.1371/journal.pone.0037591 (PMC3359301; doi:10.1371/journal.pone.0037591)
Supplement: Figure S6 — (A) Growth phenotype of the ΔpmcA::pmcA+ strain grown on YAG+CaCl2 500 mM. (B) PCR of the pmcA open reading frame. (C) PCR of the pmcB open reading frame. (PPT) [file pone.0037591.s006.ppt]

## Slide 1
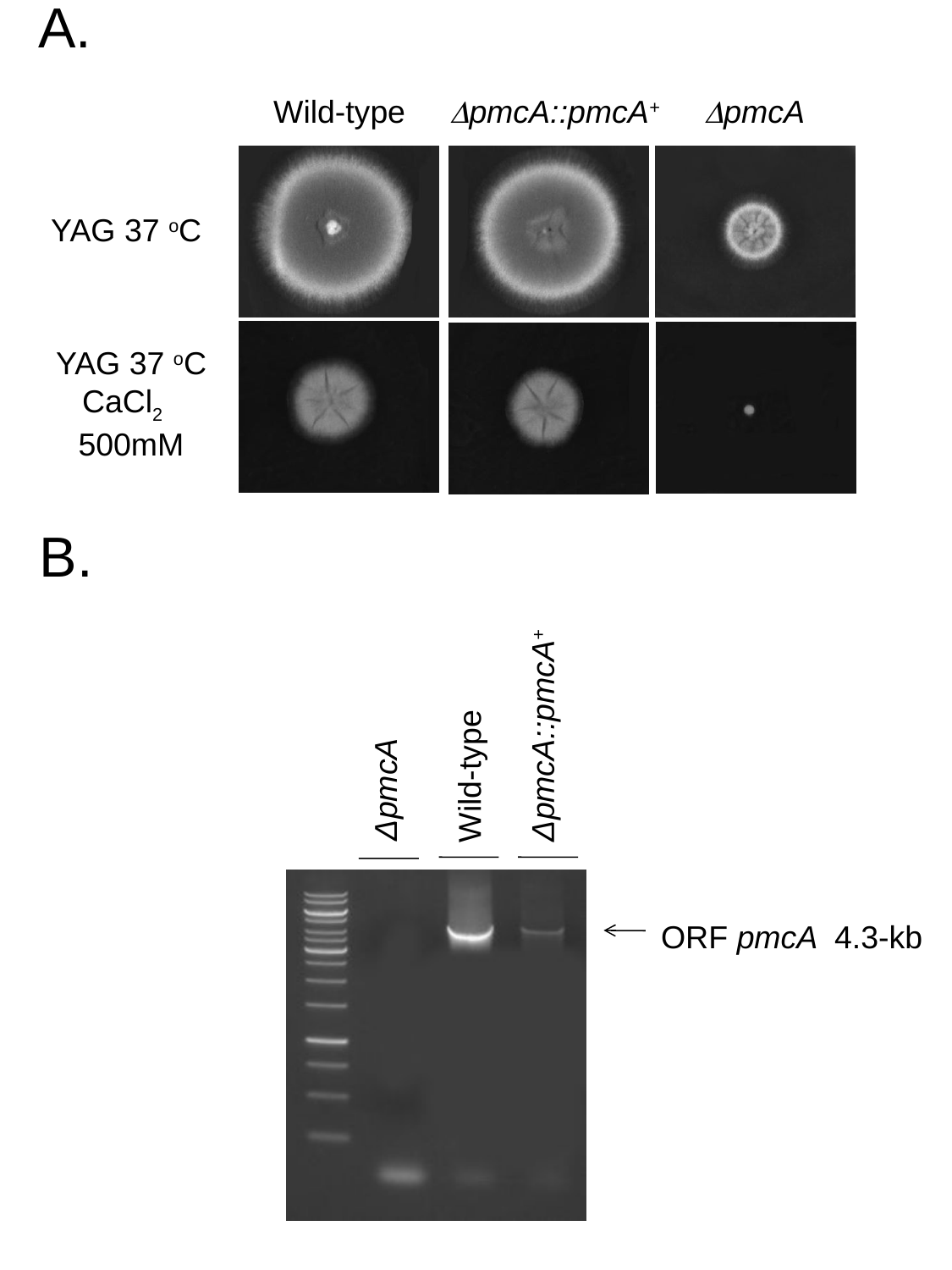

A.
 Wild-type ΔpmcA::pmcA+ ΔpmcA
YAG 37 oC
YAG 37 oC
CaCl2
500mM
B.
ΔpmcA::pmcA+
Wild-type
ΔpmcA
ORF pmcA 4.3-kb

## Slide 2
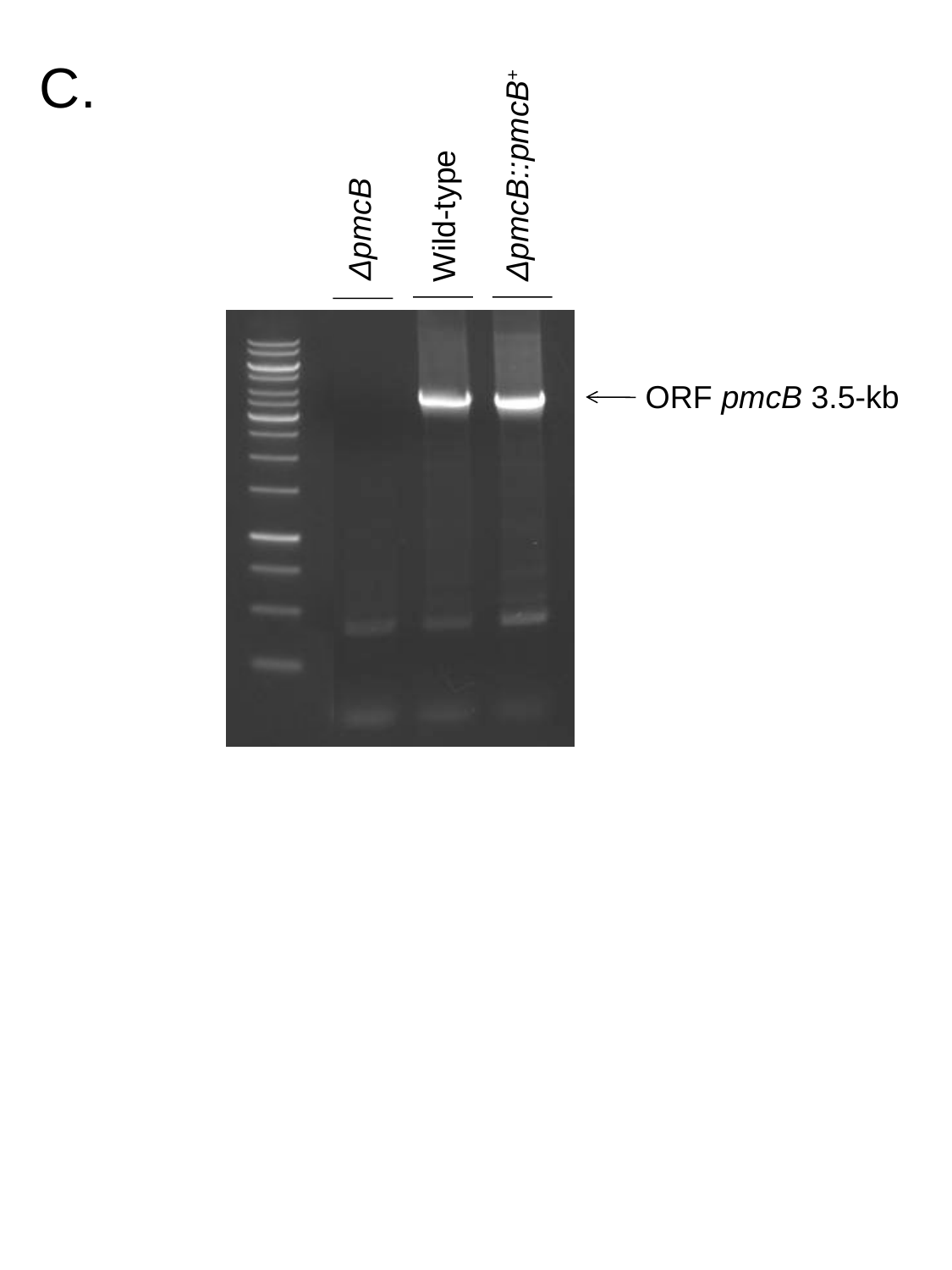

C.
ΔpmcB::pmcB+
Wild-type
ΔpmcB
ORF pmcB 3.5-kb

## Slide 3
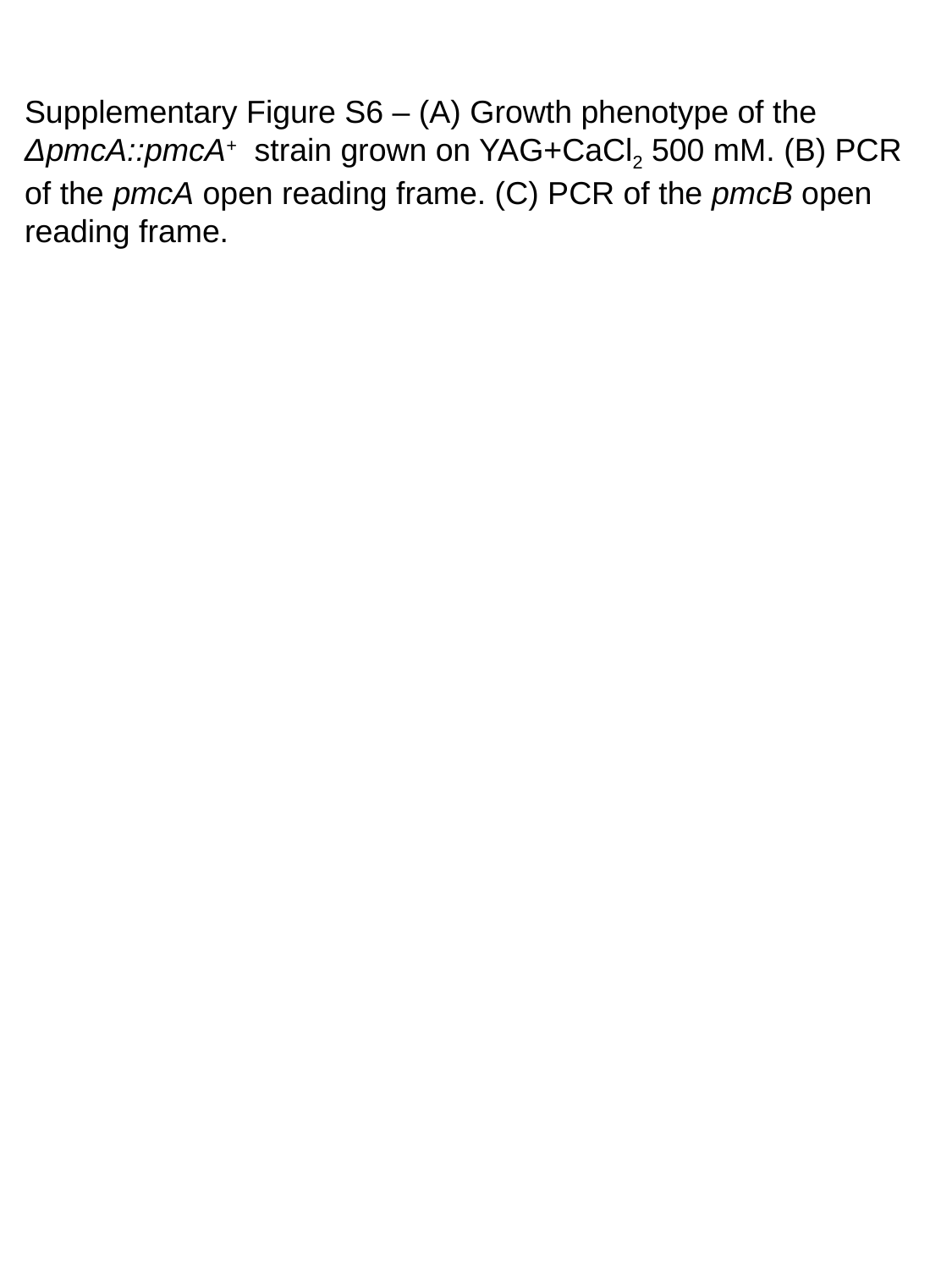

Supplementary Figure S6 – (A) Growth phenotype of the
ΔpmcA::pmcA+ strain grown on YAG+CaCl2 500 mM. (B) PCR
of the pmcA open reading frame. (C) PCR of the pmcB open
reading frame.
