# Supplementary material for: Functional Characterization of an Aspergillus fumigatus Calcium Transporter (PmcA) that Is Essential for Fungal Infection
Source: PLoS One. 2012 May 23;7(5):e37591. doi: 10.1371/journal.pone.0037591 (PMC3359301; doi:10.1371/journal.pone.0037591)
Supplement: Table S1 — List of primers and probes used in this work. (DOC) [file pone.0037591.s007.doc]

Supplementary Table S1 – List of primers and probes used in this work.

| **Name** | **Sequence** |
| --- | --- |
| **5UTR Afu pmcaC fw** | CCTGCTATCTACCTCACTGTGTGCTATG |
| **3UTR Afu pmcaC rev** | TGCCGTGCCTAGTGACGACAATTGAG |
| **pmcA 5f** | GTAACGCCAGGGTTTTCCCAGTCACGACGGACGACCTATGGGTCTGCAAC |
| **pmcA 5r** | GTGCCTCCTCTCAGACAGAATGATGGCCCAGGATTTGGAGC |
| **pmcA 3f** | GAGCATTGTTTGAGGCGAATTCATGATAATCAATGCTTGGTCATC |
| **pmcA 3r** | GCGGTTAACAATTTCTCTCTGGAAACAGCTCAGAGGGAATCAACGTGATCTAAC |
| **pmcB 5f** | GTAACGCCAGGGTTTTCCCAGTCACGACGCGTACATAGTTGCCATTACCCTTG |
| **pmcB 5r** | GTGCCTCCTCTCAGACAGAATGGTTAATATATCTCTGTCATACC |
| **pmcB 3f** | GAGCATTGTTTGAGGCGAATTCTGATTCTATACGACCCGCTCGGGCTTG |
| **pmcB 3r** | GCGGTTAACAATTTCTCTCTGGAAACAGCCCAAACTCAACTTCTAAGAAGAGTAGC |
| **pmcC AscI fw** | GGCGCGCCAATGAGTCTCAGGCTTGT |
| **pmcC PacI rv** | CCTTAATTAACATCCTGTTCTGCGTCAG |
| **pmcA1** | 5´-TGTTGCCCTGCCCCTTGTC-3´ |
| **pmcA2** | 5´-GACAAGGGGCAGGGCAACA-3´ |
| **pmcC1** | 5´-TCCGACACAGCCACGCTCT-3´ |
| **pmcC2** | 5´-AGAGCGTGGCTGTGTCGGA-3´ |
| **mPMCA-F** | 5'-CCTGGAATGTTGAAAGTAAAATTGTCACAATTTCC-3' |
| **mPMCA-R** | 5'-GGAAATTGTGACAATTTTACTTTCAACATTCCAGG-3' |
| **mPMCC-F** | 5'-GGGTGAGTCCGAACACTAACAGCTCTATGGG-3' |
| **mPMCC-R** | 5'-CCCATAGAGCTGTTAGTGTTCGGACTCACC-3' |
| **pyrG fw** | ATTCTGTCTGAGAGGAGGCACTGATGCG |
| **pyrG rev** | GAATTCGCCTCAAACAATGCTCTTCACC |
| **5’ pmcA 295 fw** | GTGTGCCGCTATCTTCTTCG |
| **5’ pmcB 295 fw** | TCTAGATAAATGATATGATACATTCATC |
| **5’ pmcC 295 fw** | CTGCCCTGATTACCGTCATG |
| **crz-exon2 attB1** | GCAGGCTTCGAAGGAGATAGAACCATGTCCCGCGGGCGTAGCAAG |
| **crz-attB2 Rv** | CAAGAAAGCTGGGTCATAGAAGTTACCGGCAGCAGAATACCC |
| **BP1** | GGGGACAAGTTTGTACAAAAAAGCAGGCTTC |
| **BP2** | GGGACCACTTTGTACAAGAAAGCTGGGTC |
| **ATT1** | TCGCGTTAACGCTAGCATGGATCTC |
| **ATT2** | GTAACATCAGAGATTTTGAGACAC |
| **pmcA 364RL** | CGGACCCTGGAAACGAACTCGTC[FAM]G |
| **pmcA 364RL/3591FU** | GGCGTGATTTCTCTGCCTGTCCGGAACTGTC |
| **pmcB 3488RL** | CGGAACTGTCGTGGAGTCGTCTTC[FAM]G |
| **pmcB 3488RL/3454FU** | TCGTCTTATGATGTGGCCTGTCA |
| **pmcC 168RL** | GACGGGCCATTTACAGCAGCTC |
| **pmcC 168RL/120FU** | CGGTCTCAGCCATAGCAACTGGAC[FAM] |
| **B tubulin 634FL** | CGAGCCCTCTCGTTCGCCAGCT[FAM]G |
| **B tubulin 663RU** | GTCGTACAGCGCCTCGTTGTCG |

* FAM: 6-carboxyfluorescein
